# Transcriptome and Co-Expression Network Analyses Identify Key Genes Regulating Nitrogen Use Efficiency in *Brassica juncea* L.

**DOI:** 10.1038/s41598-018-25826-6

**Published:** 2018-05-10

**Authors:** Parul Goel, Nitesh Kumar Sharma, Monika Bhuria, Vishal Sharma, Rohit Chauhan, Shivalika Pathania, Mohit Kumar Swarnkar, Vandna Chawla, Vishal Acharya, Ravi Shankar, Anil Kumar Singh

**Affiliations:** 1 Department of Biotechnology, CSIR-Institute of Himalayan Bioresource Technology, Palampur, 176061 (HP) India; 2 Academy of Scientific and Innovative Research, New Delhi, India; 3 ICAR-Indian Institute of Agricultural Biotechnology, PDU Campus, IINRG, Namkum, Ranchi, 834010 India

## Abstract

Nitrate is the main source of inorganic nitrogen for plants, which also act as signaling molecule. Present study was aimed to understand nitrate regulatory mechanism in *Brassica juncea* cultivars, with contrasting nitrogen-use-efficiency (NUE) *viz*. Pusa Bold (PB, high-NUE) and Pusa Jai Kisan (PJK, low-NUE), employing RNA-seq approach. A total of 4031, 3874 and 3667 genes in PB and 2982, 2481 and 2843 genes in PJK were differentially expressed in response to early, low (0.25 mM KNO_3_), medium (2 mM KNO_3_) and high (4 mM KNO_3_) nitrate treatments, respectively, as compared to control (0 mM KNO_3_). Genes of N-uptake (*NRT1*.*1*, *NRT1*.*8*, and *NRT2*.*1*), assimilation (*NR1*, *NR2*, *NiR*, *GS1*.*3*, and *Fd-GOGAT*) and remobilization (*GDH2*, *ASN2–3* and *ALaT*) were highly-upregulated in PB than in PJK in response to early nitrate treatments. We have also identified transcription factors and protein kinases that were rapidly induced in response to nitrate, suggesting their involvement in nitrate-mediated signaling. Co-expression network analysis revealed four nitrate specific modules in PB, enriched with GO terms like, “Phenylpropanoid pathway”, “Nitrogen compound metabolic process” and “Carbohydrate metabolism”. The network analysis also identified HUB transcription factors like mTERF, FHA, Orphan, bZip and FAR1, which may be the key regulators of nitrate-mediated response in *B*. *juncea*.

## Introduction

Nitrogen (N) is one of the most important nutrients for plant growth and development. Crop yield and productivity highly depend on plant nitrogen status. The crop nitrogen demand is fulfilled by applying N-fertilizers in the field. The excessive use of these fertilizers not only adds to the cost of crop production but also creates environmental pollution. Thus, for sustainable agriculture, increasing plant nitrogen use efficiency (NUE) is an important area of research. The NUE of any plant depends mainly on plant N-uptake efficiency, N-utilization efficiency and also the remobilization efficiency of nitrogen from senescing tissue to growing plant parts. The plant NUE is highly complex trait that requires coordination of several processes both at molecular and physiological levels. In agricultural soil, nitrate (NO_3_^−^) is considered as a major source of nitrogen for plants^1^. As a nutrient, nitrate is reduced to ammonium (NH_4_^+^) ions which are finally incorporated into amino acids that are vital for plant growth. As a signal, nitrate can regulate expression of several genes in plant system^2,3^. Genome-wide microarray studies in response to nitrate treatment on *Arabidopsis* has revealed significant changes in the expression of genes mainly involved in nitrate uptake and its metabolism, protein synthesis, carbohydrate metabolism and also genes involved in iron and sulfur metabolism^3,4^. Several transcriptome related studies in response to nitrogen have also been performed in maize^5^, poplar^6^, and cucumber^7^. These studies have provided valuable information to understand the N-regulatory network in plants.

Genotypic difference in terms of nitrogen use efficiency has been studied in several crops including rice^8^, wheat^9^, and soybean^10^. To study the crop N-response, it is important to compare the transcriptional responses of genotypes differing in their NUE. Till date, transcriptomic profiling of low- and high- NUE genotypes has been carried out only in very few crops. Hao *et al*.^10^ have first performed the genome-wide analysis of two soybean varieties; one tolerant and one sensitive to low N-condition and identified several genes differentially expressed between two soybean genotypes at low N-conditions. Another genome-wide transcriptional profiling has been performed in root tissues of seven sorghum genotypes (four low N-tolerant genotypes and three low N-sensitive genotypes) under low N-stress^11^. Similar studies have been conducted in barley^12^. Genome-wide transcriptional response was also studied in root tissues of two maize inbred lines differing in NUE under varying nitrate treatments using microarray approach^13^. These studies have laid a foundation for understanding the complexity of plant nitrate regulatory network at the transcriptional level. Network analysis is also one of the important approaches to study and analyze the biological data in a comprehensive manner^14^.

*Brassica juncea* (L.) Czern belongs to the Cruciferae (brassicaceae) or mustard family. *Brassica* oil seed species are the third most important oilseed crops in the world after palm oil and soybean. Oilseed *Brassica* species have been found to be less efficient in terms of their NUE as compared to cereals like, wheat, barley, rye, and oats^15^. Due to its low NUE, large amounts of N fertilizers are applied for high yield of Brassica^16^. High application rates of N fertilizers are not only costly to the farmers, but also decline crop’s NUE^17^. Genotypic differences in the efficiencies of N uptake and utilization have been reported among various cultivars of *Brassica* species^18^. These variations have also been studied in fourteen different genotypes of *B*. *juncea*^19^ and classified Pusa Bold (PB) as highest nitrogen use efficient genotype and Pusa Jai Kisan (PJK) as least nitrogen use efficient genotype of *B*. *juncea*. In a recent report, Yousuf *et al*.^20^ demonstrated that PB adapts better under elevated CO_2_ and low nitrogen conditions by upregulating several key proteins related to N metabolism as compared to PJK.

Brassica sp. seems to have a distinct regulatory mechanism of its N response. In general, most steps in nitrate assimilatory pathway are nitrate inducible^21^. By contrast, ammonium or its metabolic products exert inhibitory effects on the nitrate assimilatory pathway^22^. But in case of *Brassica napus* seedlings, it was found that nitrate was not essential for the induction of the nitrate reductase (NR) activity and NR gene expression. Moreover, ammonium supply in the absence of nitrate stimulated the NR activity in shoots more than nitrate^23^. This unusual regulatory mechanism of NR in *Brassica* seedlings indicates towards a non-canonical N-response. Therefore, present study was undertaken to understand the molecular regulatory mechanism of *B*. *juncea* in response to varying nitrate supply. Comparative physiological and global gene expression profiling of *B*. *juncea* cultivars Pusa Bold (PB) and Pusa Jai Kisan (PJK) were carried out under different nitrate conditions i.e. zero nitrate (0 mM KNO_3_), low nitrate (0.25 mM KNO_3_), medium nitrate (2 mM KNO_3_) and high nitrate (4 mM KNO_3_) at various time points. In addition, we have also performed weighted gene co-expression network analysis (WGCNA) of RNA-Seq data and identified several HUB transcription factor (TF) genes, which may act as key regulators in response to nitrate treatment.

## Results

### Effect of various nitrate concentration on growth characteristics

To investigate the effect of various nitrate concentrations on both, PB and PJK cultivars of *B*. *juncea*, growth parameters, like fresh weight and dry weight of root and shoot and length of root and shoot were recorded in seedlings after 21 days exposure to 0 mM, 0.25 mM, 2 mM and 4 mM KNO_3_ treatments (Table 1). In particular, the shoot length, root, shoot fresh and dry weight were found to be increased significantly with increasing nitrate concentration. Whereas, root length was found to be reduced with increased nitrate concentration in both the cultivars (Table 1). Interestingly, high NUE cultivar PB exhibits more increase in root and shoot dry weight than low NUE cultivar, PJK in response to nitrate treatments (Table 1).

**Table 1:** Effect of nitrate concentration on growth characteristics.

Nitrate supply	Genotype	Root fresh weight (g)	Shoot fresh weight (g)	Root dry weight (g)	Shoot dry weight (g)	Root length(cm)	Shoot length (cm)
0 mM KNO_3_	PB	0.41 ± 0.01a	0.98 ± 0.08a	0.021 ± 0.001	0.11 ± 0.002a	20.625 ± 1.88a	9.66 ± 0.47a
	PJK	0.21 ± 0.03b	0.6 ± 0.07b	0.022 ± 0.001	0.05 ± 0.004b	23 ± 2.12b	6.41 ± 0.38b
0.25 mM KNO_3_	PB	0.51 ± 0.01	1.04 ± 0.09a	0.051 ± 0.001	0.12 ± 0.009a	18.82 ± 1.13	11.57 ± 0.52a
	PJK	0.52 ± 0.001	0.81 ± 0.02b	0.056 ± 0.004	0.063 ± 0.004b	19.74 ± 2.11	9.76 ± 0.55b
2 mM KNO_3_	PB	0.66 ± 0.02	1.21 ± 0.10	0.069 ± 0.002a	0.18 ± 0.003a	16.8 ± 1.53a	12.36 ± 0.23
	PJK	0.61 ± 0.04	1.13 ± 0.07	0.053 ± 0.001b	0.138 ± 0.001b	18.28 ± 2.8b	12.13 ± 0.55
4 mM KNO_3_	PB	1.11 ± 0.016a	1.42 ± 0.12	0.082 ± 0.004a	0.19 ± 0.004a	16.3 ± 0.44	17.63 ± 0.63
	PJK	0.7 ± 0.011b	1.33 ± 0.05	0.070 ± 0.003b	0.169 ± 0.022b	17 ± 1.76	17.54 ± 1

Root fresh weight (g), shoot fresh weight (g), root dry weight (g), shoot dry weight (g), root length (cm) and shoot length (cm) were measured 21 day after giving various nitrate treatment (0.25 mM KNO_3_, 2 mM KNO_3_ and 4 mM KNO_3_) in PB and PJK are shown. Data indicates mean ± S.E (n = 6). For control conditions, equal amount of KCl was supplied to the plants. Values labelled with different lowercase letters indicate significance level of difference with in the cultivars with in the treatment (p < 0.05, using DMRT).

### Effect of various nitrate concentrations on leaf nitrate reductase activity (NRA) and nitrate ion content

**Figure 1:**
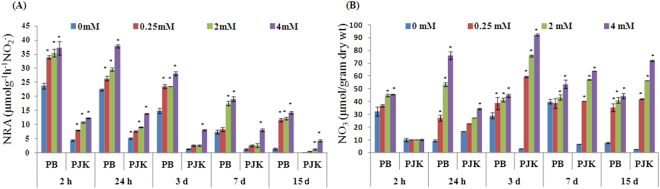
Effect of nitrate concentration on NRA and nitrate content: Leaf nitrate reductase activity (NRA) (**A**) and nitrate content (**B**) was measured in PB and PJK after 2 h, 24 h, 3 d, 7 d and 15 d of various nitrate treatments (0.25 mM KNO_3_, 2 mM KNO_3_ and 4 mM KNO_3_). For control conditions (0 mM KNO_3_), equal amount of KCl was supplied to the plants. The vertical bars indicate mean ± S.E (n = 3). Asterisk on the top of the bar indicates level of significance (*p-value < 0.05, student’s t-test).

The leaf nitrate reductase activity (NRA) and nitrate content were also measured in both the cultivars at 2 h, 24 h, 3 d, 7 d and 15 d after various nitrate treatments i.e. 0 mM KNO_3_ (control), 0.25 mM KNO_3_ (low nitrate), 2 mM KNO_3_ (medium nitrate) and 4 mM KNO_3_ (high nitrate). The NRA was found to be increased (>1.5 fold) with increasing nitrate concentrations at each time point in both the cultivars w.r.t control (Fig. 1A). In case of PB, the maximum NRA was observed after 24 h of nitrate treatment w.r.t control and thereafter the NRA decreased gradually in control as well as treated samples (Fig. 1A), whereas in case of PJK, the maximum NRA was observed after 3 d of nitrate supply (Fig. 1A). The NRA of PB at low nitrate condition ranged from 1.42 to 7.0 fold higher than control at respective time points. Interestingly, the NRA of PB in control as well as treated samples was found to be several folds (1.3 to 17.6 fold) higher as compared to NRA in respective samples of PJK at all the time points.

The accumulation of nitrate ions was found to be increased with increasing nitrate concentration w.r.t control (Fig. 1B). The maximum nitrate ion content was found under 4 mM KNO_3_ treatment at each time point in both the cultivars (Fig. 1B). In case of PB, the accumulation of nitrate ions was found to be high at 24 h of nitrate treatment w.r.t control. However, in case of PJK, the nitrate ions were found to be accumulated more at 3 d of nitrate treatment w.r.t control (Fig. 1B).

### RNA-seq analysis of *B. juncea* cv. PB and PJK in response to nitrate treatments

In order to study the global transcriptional response of *B*. *juncea* cv. PB and PJK in response to various nitrate treatments, RNA-seq analysis of 42 different samples (21 samples from each cultivar) that were subjected to various nitrate treatments (0 mM KNO_3_, 0.25 mM KNO_3_, 2 mM KNO_3_ and 4 mM KNO_3_) at six different time points (20 min, 2 h, 12 h, 24 h, 3 d and 7 d) was performed (Supplementary Table S1). A total of 542,339,262 raw paired-end reads were generated, out of which, a total of 446,580,418 clean reads were used for assembly (Supplementary Table S2). The *de novo* assembly was performed using SOAPdenovo assembler, which was run at different k-mer size ranging from 19 to 29 mers with read-length of 33 bp. A total of 91,765 transcripts with an average length of 903.39 bp, N50 value 1,427 and average coverage of 160× were obtained after assembly (Supplementary Table S3). After gap filing, hierarchical clustering and removing mis-assembled transcripts, a total of 46,556 assembled transcripts were obtained that were used for further analysis (Supplementary Table S3). The homology search of correctly assembled sequences was performed using BLASTX against non-redundant protein database of NCBI with E-value 10^−5^. Out of 46,556 transcripts, 41,278 were found to have significant BLASTX hits, whereas no hits were found for 5,278 transcripts. In order to validate the assembled sequences of *B*. *juncea*, BLASTN was performed at an E-value 10^−5^ using assembled transcripts as a database. A total of 5,518 ESTs of *B*. *juncea* were available in NCBI database, out of which 5,111 (92.62%) ESTs have significant hits with assembled transcripts. A total of 1,449 ESTs (26% of total ESTs) were found to have 100% width coverage. A total of 3,207 ESTs (58% of total ESTs) were found to have width coverage ≥90% and 4,807 (87% of total ESTs) ESTs have width coverage ≥50%.

Initially, *de novo* transcriptome assembly was performed, which was used for expression analysis. Since, *B*. *juncea* genome is recently published^24^, transcriptome assembly was performed using *B*. *juncea* genome as reference, and the assembled data was utilized for expression analysis. Systematic comparison of expression profiles as obtained after *de novo* and reference-based assemblies was done in order to assess the degree of agreement between the two approaches. We observed that 70–80% genes exhibited similar expression pattern when the *de novo* transcriptome data was compared with recently published *B*. *juncea* genome. Also, the *de novo* assembled transcriptome sequence displayed an overall good agreement (82.5%) with the coding sequences reported for the reference genome.

### GO and KEGG enrichment analysis

**Figure 2:**
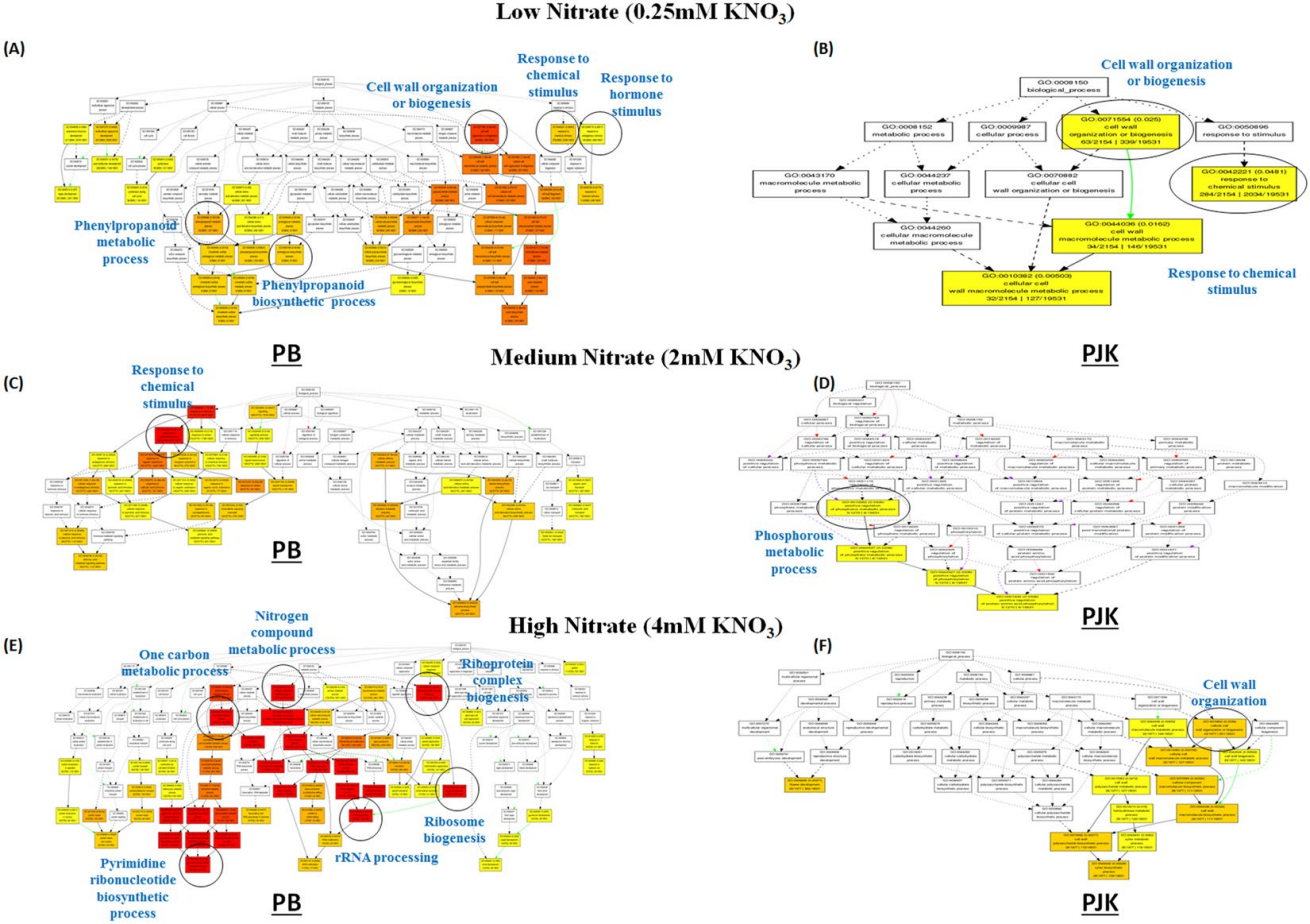
GO biological process enrichment of PB and PJK in response to low (0.25 mM KNO_3_), medium (2 mM KNO_3_) and high nitrate treatment (4 mM KNO_3_): Hierarchical tree graph of overrepresented GO terms in biological function category in response to low (**A**,**B**), medium (**C**,**D**) and high nitrate (**E**,**F**) treatments. Boxes in the graph represent GO terms with their GO ID, term definition and statistical information. Significant GO terms (p ≤ 0.05) are marked with color and non-significant GO terms are shown in white boxes. The degree of color saturation of a box is positively correlated to the enrichment level of the term. Solid, dashed, and dotted lines represent two, one and zero enriched terms at both ends connected by the line, respectively. The rank direction of the graph is set to from top to bottom.

The correctly assembled sequences were functionally annotated using Annot8r program. All the assembled sequences were compared against UniProt database with an E-value 10^−1^. The various functional categories named as GO (Gene Ontology), EC (Enzyme Classification) and KEGG (Kyoto Encyclopedia of Genes and Genomes) were then assigned to the sequences based on the highest top scoring hits (Supplementary Fig. S1). Gene Ontology classification was predicted for all 25,400 best group representative unigenes in order to characterize their functionality. Out of 25,400 transcripts, a total of 20,884 transcripts were annotated under GO term. The GO biological terms, related to metabolic processes and response to stimulus were most represented terms (Supplementary Fig. S2A) in overall transcriptome data, irrespective of the nitrate conditions. Additionally, the GO molecular terms related to binding, catalytic and transferase activities were found to be most enriched in overall data (Supplementary Fig. S2B). The GO biological terms like, response to chemical stimulus (GO: 0042111; p-value: 0.00491), and response to hormone stimulus (GO: 0009725; 0.00171) were found to be significantly enriched in PB in response to early nitrate treatment (0.25 mM KNO_3_, 20 min) (Fig. 2A). Additionally, several GO biological terms involved in phenylpropanoid metabolic process (GO: 0009698; 0.000199), phenylpropanoid biosynthetic process (GO: 0009699; 0.000621) and cell wall organization or biogenesis (GO: 0071554; 3.28e-08) were also found to be significantly enriched (Fig. 2A). However, in case of PJK (0.25 mM KNO_3_, 20 min), GO biological terms like, cell wall organization or biogenesis (GO: 0071554; 0.025) and response to chemical stimulus (GO: 0042221; 0.0481) were only significantly enriched GO terms (Fig. 2B). Under medium nitrate treatment (2 mM KNO_3_, 2 h), the GO biological terms like response to stimulus (GO: 0050898; 1.7e-0.9), response to nitrate (GO: 0010167; 9.83e-06) was significantly enriched in PB (Fig. 2C), whereas in PJK the only significantly enriched GO biological term was related to phosphorous metabolism (GO: 00105662; 0.0338) (Fig. 2D). At, high nitrate treatment (4 mM KNO_3_, 2 h) pyrimidine nucleotide metabolic process (GO: 0006220; 2.86e-27), nitrogen compound metabolic process (GO: 0006807; 3.17e-19), ribosome biogenesis (GO: 0042254; 3.01e-16), ribonucleoprotein complex biogenesis (GO: 0022613; 1.89e-12) and one-carbon metabolic process (GO: 0006730; 9.23e-50) were found to be enriched in PB (Fig. 2E). However, in case of PJK, no such GO terms were found to be enriched in response to an early high nitrate treatment (4 mM KNO_3_, 2 h) (Fig. 2F). The detailed GO enrichment analysis of the DEGs in comparative conditions of PB vs PJK was also carried out in response to early nitrate treatment (i.e 20 min or 2 h) (Supplementary Table S4).

We assigned 12,575 unigenes to 162 KEGG pathways. Top-20 KEGG pathways in our transcriptome data are shown in Supplementary Fig. 2C. The plant pathogen interaction, plant hormone signal transduction and ribosome (539, 4.29%) were highly represented KEGG pathway in overall data. The genes involved in the pathway of RNA transport, spliceosome, protein processing in endoplasmic reticulum, starch and sucrose metabolism, ubiquitin mediated proteolysis, tight junction and ribosome biogenesis in eukaryotes were also among top-20 KEGG category. In addition, many genes corresponding to pathways such as, carbohydrate biosynthesis and metabolism including glycolysis/gluconeogenesis, oxidative phosphorylation, TCA cycle, pentose phosphate pathway and galactose metabolism were also present. The pathways involved in secondary metabolite biosynthesis included stilbenoid, diarylheptanoid and gingerol biosynthesis, diterpenoid biosynthesis, flavones and flavonol biosynthesis, indole alkaloid biosynthesis, anthocyanin biosynthesis, sesquiterpenoid biosynthesis were also found under KEGG category. The detailed KEGG enrichment analysis was also performed for DEGs between the cultivars (PB vs PJK) at early nitrate treatments (Supplementary Table S4).

### Differential gene expression analysis

In order to identify the differentially expressed genes (DEGs) in response to various nitrate treatments in both the genotypes of *B*. *juncea*, a total of 30 comparative conditions were taken in to consideration (Supplementary Table S5). For identification of differentially expressed transcripts, those with relative fold change two or above were considered as significant DEGs. All the significant DEGs were identified under nitrate treated condition w.r.t. control at each time point in both the genotypes (Supplementary Table S5). Most of the nitrate responsive genes were found to respond after 20 min or 2 h of nitrate supply. Therefore, we restricted further analysis to six comparative conditions named as C1, C2, C3 and C16, C17 and C18 from PB and PJK, respectively. Interestingly, it was found that a total of 4031, 3874 and 3667 genes in PB and 2981, 2480 and 2842 in PJK were upregulated in comparative conditions C1, C2, C3 and C11, C12 and C13, respectively. Higher number of upregulated genes at early nitrate treatment in high NUE cultivar PB indicates that nitrate as a signal regulates the expression of majority of genes in PB. These early inducible nitrate responsive genes in PB are listed in Supplementary Table S6. Additionally, DEGs between the cultivars (PB vs. PJK) under similar nitrate conditions were also identified (Supplementary Table S7). A total of 6396, 4378, 5731 genes were upregulated and 3311, 2083, 2332 genes were downregulated in response to early low, medium and high nitrate treatment in PB, when compared with the respective condition of low NUE cultivar, PJK.

### Involvement of transcription factors and protein kinases in response to an early (20 min), low nitrate treatment (0.25 mM KNO_3_)

**Figure 3:**
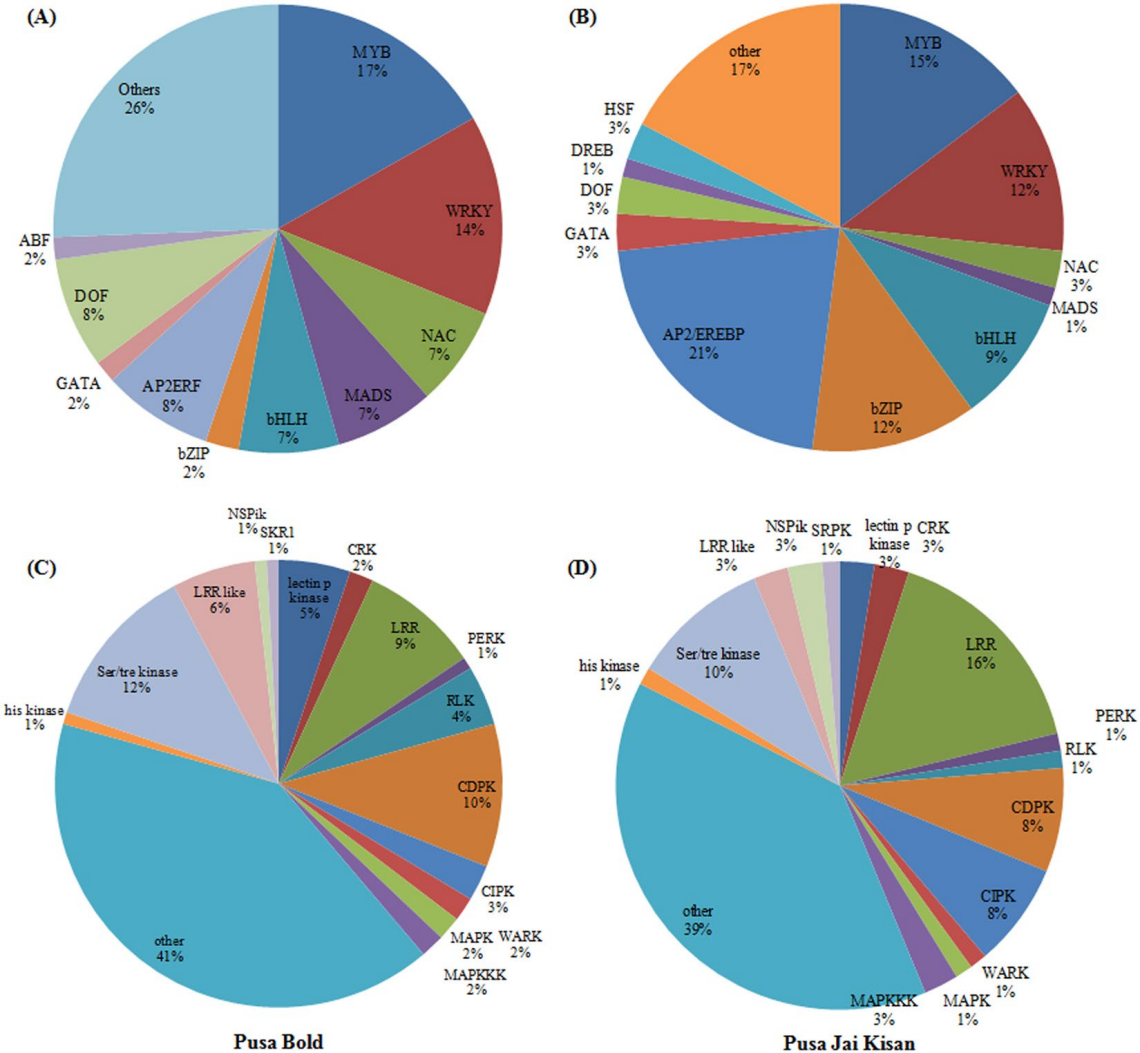
Plot showing transcription factor families (**A**,**B**) and protein kinases (**C**,**D**) found to be induced in response to early (20 min) low nitrate treatment (0.25 mM KNO_3_) in PB and PJK on the basis of their transcript abundance.

Transcription factors (TFs) and protein kinases play important roles in plant signal transduction pathways. The Top-20 TF families on the basis of their transcript abundance in complete RNA-seq data are shown in Supplementary Fig. S3. We have also identified several TF families and protein kinases that were found to be upregulated in response to an early (20 min) low nitrate treatment (0.25 mM KNO_3_). In PB, the transcripts encoding MYB (17%) and WRKY (14%) transcription factors were found to be highly induced at low nitrate treatment (0.25 mM KNO_3_) (Fig. 3A). However, in case of PJK, the transcripts encoding AP2/EREBP (21%) and MYB (15%) transcription factors were found to be highly induced (Fig. 3B). A comparison of expression profile of fifteen protein kinase families, mainly Lectin protein kinase, CRK (Cystein rich receptor like protein kinase), LRR (Leucine-rich receptor like protein kinases), PERK (Proline-rich extension-like receptor protein kinase), RLK (Receptor like kinase), CDPK (Calcium-dependent protein kinases), CIPK (CBL-interacting protein kinases), WARK (Wall-associated receptor like Kinases), MAPK (Mitogen activated protein kinase), MAPKK, Histidine kinase, Ser/thr kinase, LRR like, NSPiK (NSP-interacting kinases) and SKR1 was also performed in response to an early low nitrate treatment in both the cultivars (Fig. 3C,D). In PB, Ser/thr, CDPK, LRR, LRR like, lectin protein kinases (Fig. 3C) and in PJK, LRR, Ser/thr, CIPK, CDPK, MAPKK were top-5 overrepresented kinase classes that were found to be early-responsive (20 min) to low nitrate treatment (Fig. 3D).

### Differential expression behavior of genes involved in N-uptake and assimilation and carbohydrate metabolism in response to nitrate treatment

**Figure 4:**
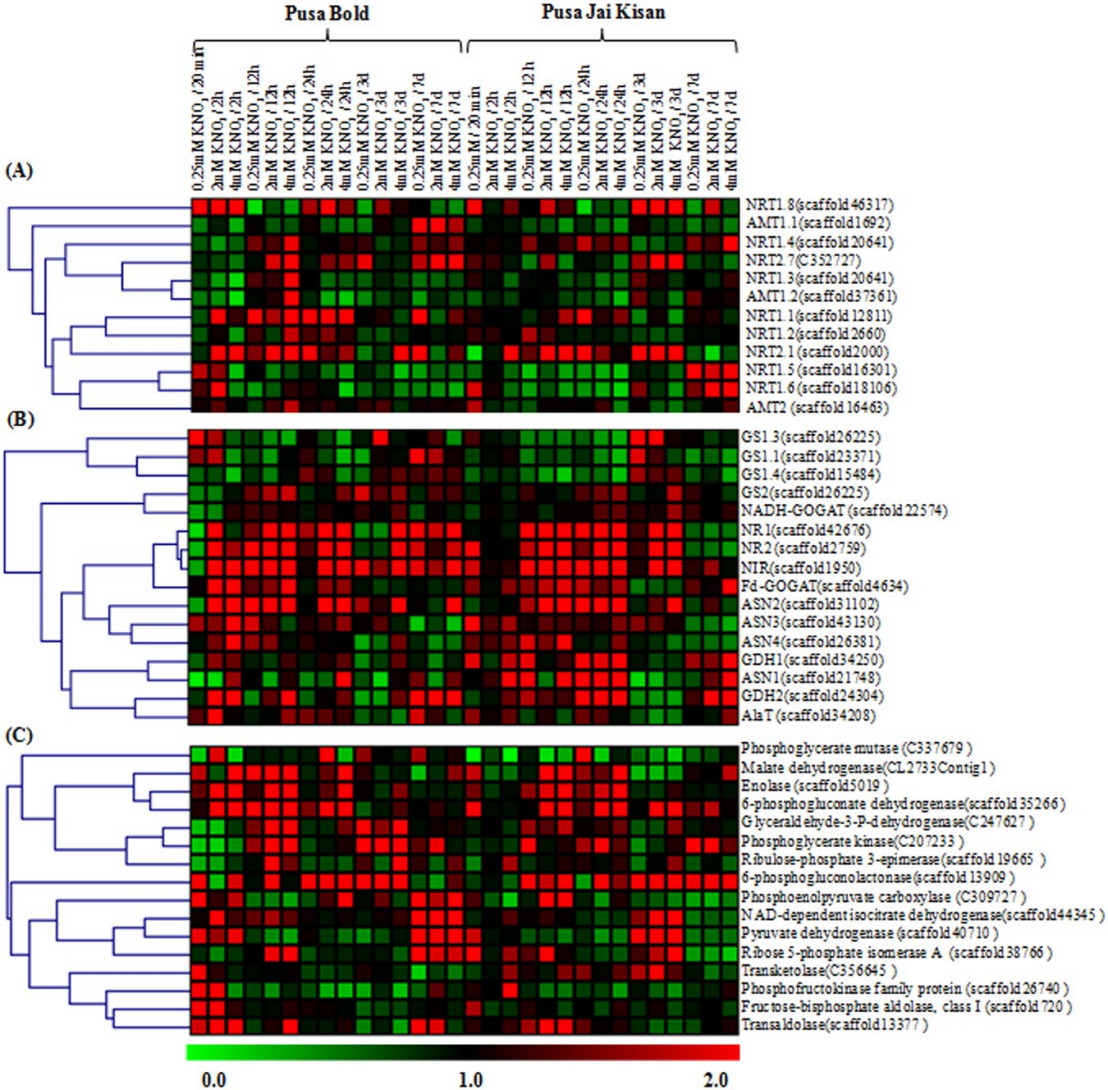
Heat map showing the relative expression of genes of N-uptake (**A**), N-assimilation (**B**) and genes involved in carbohydrate metabolism (**C**) in PB and PJK with respect to control sample of each time point in response to various nitrate treatments. The vertical bar indicates relative expression ratio where red, black and green represents upregulation, no change and downregulation in transcripts level.

A total of 28 genes involved in N-uptake, assimilation and remobilization have been identified from the transcriptome data (Supplementary Table S8). Their expression analysis revealed that majority of genes were differentially expressed in both the cultivars in response to nitrate treatment (Fig. 4A,B). Early upregulation (20 min or 2 h) of genes encoding for nitrate transporters like NRT1.1 (scaffold2660), NRT1.8 (scaffold46317) and NRT2.1 (scaffold20006) and various isoforms of genes encoding for enzymes of N-assimilation, like nitrate reductase (NR; scaffold42676, scaffold2759), nitrite reductase (NiR; scaffold1950), glutamine synthetase (GS; scaffold26225, scaffold23371), glutamate synthase (GOGAT; scaffold4634), glutamate dehydrogense (GDH, scaffold24304) and asparagine synthetase (ASN, scaffold31102, scaffold43130) was observed in PB (Fig. 4A,B). However in case of PJK, majority of genes encoding for enzymes involved in nitrate assimilation were found to be induced at later stages (Fig. 4A). Early induction of some of the genes encoding for nitrate reductase (NR2; scaffold2759) and nitrite reductase (NiR; scaffold1950) was observed at 20 min of nitrate supply in PJK but their expression was found to be immediately downregulated at later time points (Fig. 4B). Several genes of carbohydrate metabolism, like phosphofructokinase (scaffold26740), fructose-bisphosphate aldolase (scaffold720), 6-phosphogluconolactonase (scaffold13909), transketolase (C356645), transaldolase (scaffold13377) and phosphoenolpyruvate carboxylase (C309727) were found to be upregulated rapidly (20 min) in PB under low nitrate treatment (Fig. 4C). However, in PJK, only 6-phosphogluconate dehydrogenase (scaffold35266) and ribose 5-phosphate isomerase A (scaffold38766) were found to be upregulated rapidly (20 min) after nitrate supply.

To confirm the validity of RNA-seq data, eleven differentially expressed genes that are involved in processes like N-uptake (*NRT1*.*1*, *NRT1*.*2*, *NRT1*.*3, NRT1*.*4*, *AMT1*.*1*, *AMT1*.*2*, *AMT2*), assimilation (*NR1*, *NR2*, *GS1*.*3*) and remobilization (*GDH1*) were selected for qRT-PCR analysis. The gene expression trend of qRT-PCR data was in accordance with RNA-seq data; however, some samples show obscure correlation in the expression observed by FPKM and qRT-PCR value (Supplementary Fig. S4). Pearson correlation coefficient between fold change observed through FPKM values and qRT-PCR was found to be significant (r = 0.76, p-value ≤ 0.0001) indicating the reliability of our RNA-seq data.

### Weighted gene co-expression network analysis

In order to get insight of differential behavior of both the cultivars in response to nitrate treatment, co-expression network analysis was performed. The dataset for network construction consists of a total of 42 samples (21 samples from each cultivar). Filtering and pre-processing of the data led to elimination of sample 17 (PB, 4 mM KNO_3_, 3 d) as outlier from PB dataset (Supplementary Fig. S5). A total of 25,239 common genes between both datasets were used to construct two weighted networks. Positive correlation and significant p-values of average gene expression (cor = 1, p ≤ 1e-200) (Supplementary Fig. S6A) and overall connectivity (cor = 0.61, p ≤ 1e-200) (Supplementary Fig. S6B) ensured the comparability of these datasets. Evaluation of network topology was carried out using pickSoftThreshold function and appropriate power (Supplementary Figs S7, S8). Hierarchical clustering based on DisTOM identified a total of 29 and 35 modules in PB (Supplementary Fig. S9A) and PJK (Supplementary Fig. S9B), respectively. Out of these modules, 6 in PB and 3 in PJK were found to be significantly (p-value ≤ 0.05) up- or down-regulated while integrating nitrate treatments data. Module assignments were performed for test dataset (PJK) using reference dataset of PB based on significant match of overlap genes between both datasets (Supplementary Fig. S10).

### GO enrichment analysis of significant nitrate responsive modules

**Figure 5:**
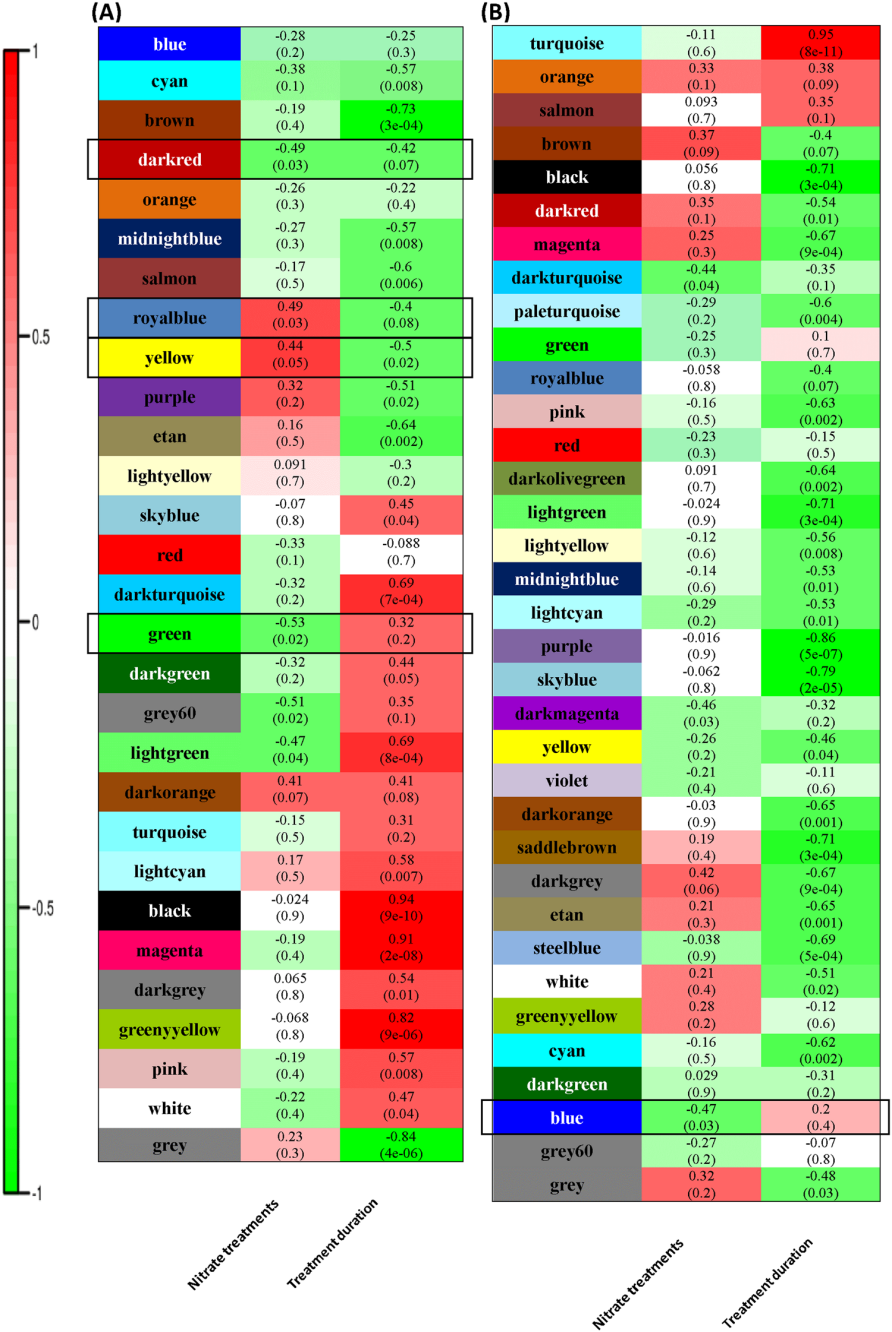
Enriched nitrate regulated modules in PB (**A**) and PJK (**B**). The module highlighted with black border box represents significant enriched module aassociated with nitrate response during enrichment analysis and selected for further analysis.

The number of enriched modules were reduced to four in PB (module darkred, green, royalblue and yellow) and one in PJK (module blue), based on the presence of at least one significant term directly associated with nitrate response during enrichment analysis (Fig. 5). The GO terms enriched in these modules are listed in Table 2 and genes from respective modules of PB and PJK are listed in Supplementary Table S9. The Darkred module of Pusa Bold was found to be significantly downregulated in response to nitrate treatment (r = −0.49 and p-value = 0.03) (Fig. 5A). A total of 198 genes were clustered in this module (Supplementary Table S9). The module was enriched with significant GO terms like cellular process (GO: 0009987), secondary metabolic process (GO: 0006725), Phenypropanoid biosynthetic process (GO: 0009699) and cellular amino acid derivative biosynthetic process (GO: 0042398) (Table 2). The green module of Pusa Bold was also found to be significantly downregulated (r = −0.53, p-value = 0.02) in response to nitrate treatment (Fig. 5A). A total of 493 genes were clustered in this module (Supplementary Table S9). The module contains several significant GO terms such as cellular process (GO: 0009987), nitrogen compound metabolic process (GO: 0006807), post-embryonic development (GO: 0009791), and metabolic process (GO: 0008152) (Table 2). The Royal blue of Pusa Bold was found to be significantly upregulated in response to nitrate treatment (r = 0.49, p-value = 0.03) (Fig. 5A). A total of 224 genes were clustered in this module (Supplementary Table S9). The module was enriched with several significant GO terms such as nitrogen compound metabolic process (GO: 0006807), cellular nitrogen compounds metabolic process (GO: 0034641), response to stimulus (GO: 0050896), glucose metabolic process (GO: 0006006). The Yellow module of Pusa Bold was also found to be significantly upregulated in response to nitrate treatment (Fig. 5A). A total of 713 genes were clustered in this module (Supplementary Table S9). The major GO terms include translation (GO: 0006412; 4.3e-91), cellular protein metabolic process (GO: 0044267; 3.08e-53), gene expression (GO: 0010467; 7.89e-55), ribosome biogenesis (GO: 0042254; 6.66e-48), protein metabolic process (GO: 0019538; 2.12e-47), cellular macromolecule biosynthetic process (GO: 0034645; 6.14e-47), ribonucleoprotein complex biogenesis (GO: 0022613; 7.51e-47), ncRNA processing (GO: 0034470; 3.14e-09), purine ribonucleoside monophosphate biosynthetic process (GO: 0009168; 0.000442), and purine nucleoside monophosphate biosynthetic process (GO: 0009127; 0.000442). The only module of Pusa Jaikisan that was found to be significantly downregulated in response to nitrate treatment was blue module (r = −0.47, p-value = 0.03) (Fig. 5B). A total of 4,237 genes were clustered in this module (Supplementary Table S9). The significant GO terms found to be enriched under this module was found to be associated with highly enriched terms like response to stimulus (GO: 0050896), cellular carbohydrate metabolic process (GO: 0044262) cellular nitrogen compound metabolic process (GO: 0034641), sulfur compound metabolic process (GO: 0044272). Taken together, these findings suggest that both the cultivars of *B*. *juncea* employ distinct transcriptome behavior in response to nitrate treatments in addition to some commonness.

**Table 2:** Table representing enriched GO terms in darkred, green, royalblue, yellow modules of PB and blue module of PJK.

GO terms	Description	p-value
**Darkred module**		
GO: 0009987	Cellular process	0.0297
GO: 0006725	Secondary metabolic process	0.0058
Go: 0009699	Phenylpropanoid biosynthetic processs	0.0121
GO: 0042398	Cellular amino acid derivative biosynthetic process	0.0266
**green module**		
GO: 0009987	Cellular process	0.0088
GO: 0006807	Nitrogen compound metabolic process	0.0031
GO: 0009791	Post-embryonic development	0.0006
GO: 0008152	Metabolic process	0.0137
**Royalblue module**		
GO: 0006807	Nitrogen compound metabolic process	0.0008
GO: 0034641	Cellular nitrogen compound metabolic process	0.0011
GO: 0050896	Response to stimulus	0.0002
GO: 0006006	Glucose metabolic process	0.0096
**Yellow module**		
GO: 0034641	Cellular nitrogen compound metabolic process	0.0233
GO: 0043170	Macromolecule metabolic process	3.25e-37
GO: 009058	Biosynthetic process	3.02e-43
GO: 0022613	Ribonucleoprotein complex biogenesis	7.51e-47
**blue module**		
GO: 0050896	Response to stimulus	3.14e-16
GO: 0044262	Cellular carbohydrate metabolic process	5.34e-10
GO: 0034641	Cellular nitrogen compound metabolic process	2.19e-14
GO: 0044272	Sulfur compound biosynthetic process	3.89e-07

### Identification of HUB transcription factors as putative master regulators in significant nitrate responsive modules

**Figure 6:**
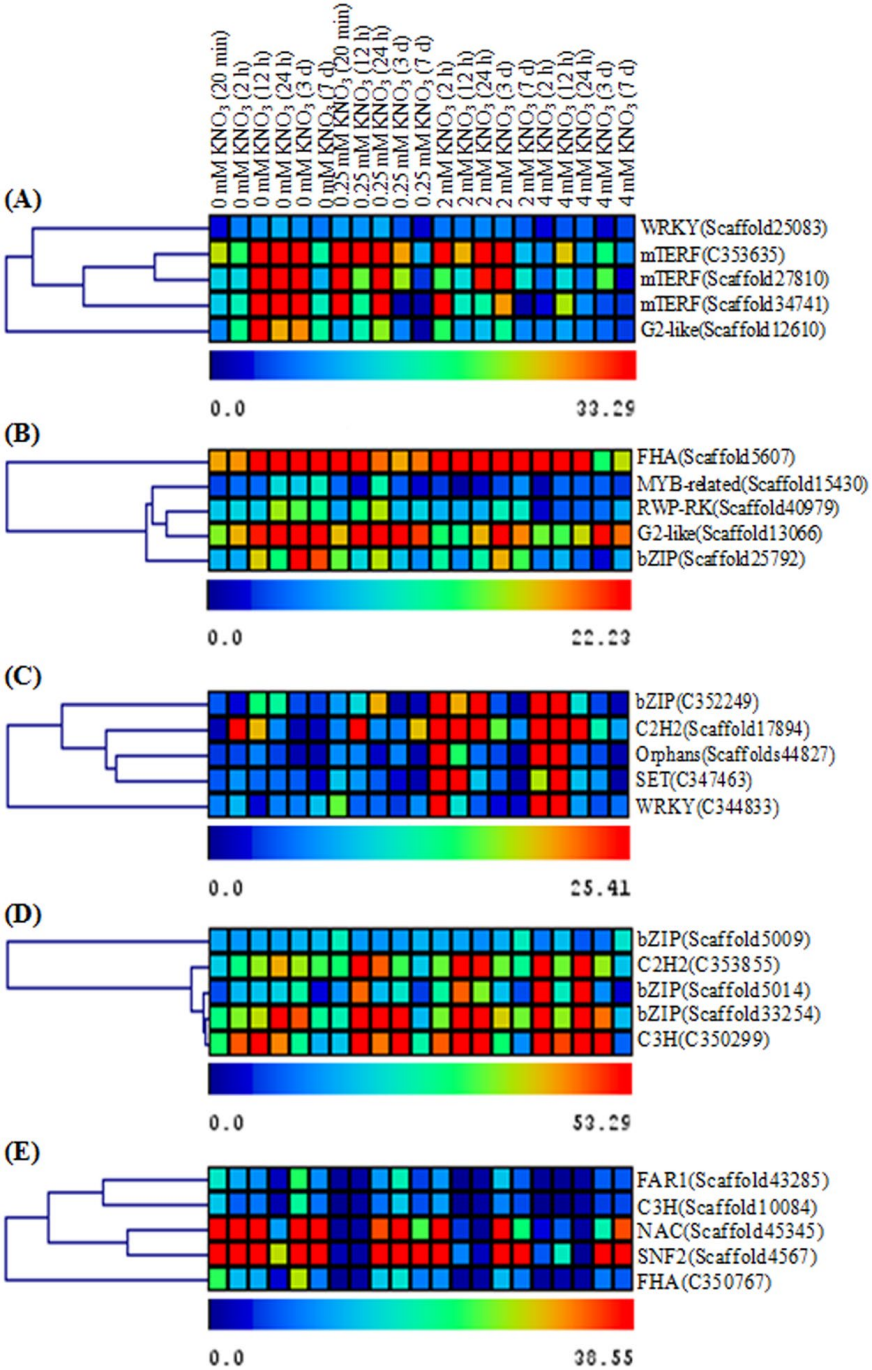
Nitrate regulatory modules and HUB TF genes in *B*. *juncea*: Four nitrate regulatory modules in PB named as Darkred, green, Royalblue, yellow (**A**–**D**) and one in PJK named as blue module (**E**) are identified. Based on the connectivity (K) and gene significance (GS), top-5 HUB TFs are identified in each module. The differential expression patterns of these HUB TFs under control and treated conditions are shown.

The N-regulatory network in plants is highly complex and TFs can act as potential regulators in controlling gene expression, hence we have identified the transcription factors (TFs) as central genes (hub genes) in nitrate responsive modules based on their scaled connectivity (K) and gene significance (GS). The Top-five putative HUB TF genes in significant nitrate responsive modules and their relative expression profiles are shown in Fig. 6; Table 3. Among top-5 HUB TF genes in darkred module, three contigs (C353635, scaffold27810, scaffold34741) were found to encode mTERF TFs, out of which C353635 contig has maximum scale connectivity (K = 1), followed by scaffold27810 (K = 0.97) and scaffold34741 (K = 0.95). The WRKY (scaffold25083, K = 0.96) and G2-like TF (scaffold12610, K = 0.93) are also among the top-5 HUB TF genes in darkred module (Fig. 6A, Table 3). In green module, HUB TF belonging to FHA TF family (scaffold560) was found to have maximum connectivity (K = 0.99) followed by RWP-RK (scaffold40979, K = 0.96) MYB-related (scaffold15430, K = 0.95), G2-like (scaffold13066, K = 0.94) and bZIP (scaffold25792, K = 0.93) (Fig. 6B, Table 3). In royalblue module, the orphan TF (scaffold44827) was found to have maximum connectivity (K = 1), followed by C2H2 (scaffold17894, K = 0.98), bZIP TF (C352249, K = 0.97), SET (C347463, K = 0.95) and WRKY (C344833, K = 0.93) (Fig. 6C, Table 3). The top ranking TF in yellow module was bZIP TF (scaffold33254, K = 0.99), C2H2 (C353855, K = 0.98), bZIP (C352249, 0.97), (Fig. 6D, Table 3). In blue module of PJK, the FAR1 (scaffold43285, K = 0.99), C3H (scaffold10084, K = 0.99), NAC (scaffold45345, K = 0.99), SNF2 (scaffold4567, K = 0.98) and FHA (C350767, K = 0.98) were top-5 highest ranking HUB TF genes (Figs 6E, Table 3).

**Table 3:** Top-5 HUB TFs identified in nitrate responsive modules of *B*. *juncea* cv.

TF Family	TSIDs	ATID	K_PB_Darkred	GS_PB_Darkred
**Darkred Module (Pusa Bold)**				
mTERF	C353635	AT1G03940.1	1	0.502264892
mTERF	scaffold27810	AT3G29590.1	0.973730396	0.441392809
WRKY	scaffold25083	AT4G30935.1	0.967432093	0.423912887
mTERF	scaffold34741	AT1G03940.1	0.954885337	0.407510167
G2-like	scaffold12610	AT1G80050.1	0.939802622	0.473269354
**TF Family**	**TSIDs**	**ATID**	**K_PB_Green**	**GS_PB_Green**
**Green Module (Pusa Bold)**				
FHA	scaffold560	AT2G21300.2	0.990369339	0.418543425
RWP-RK	scaffold40979	AT1G76350.1	0.964369287	0.682719805
MYB-related	scaffold15430	AT1G01520.1	0.955176601	0.440784753
G2-like	scaffold13066	AT3G24120.1	0.948521958	0.513756988
bZIP	scaffold25792	AT1G75390.1	0.937654789	0.474198621
**TF Family**	**TSIDs**	**ATID**	**K_PB_Royalblue**	**GS_PB_Royalblue**
**Royalblue Module (Pusa Bold)**				
Orphans	scaffold44827	AT1G78050.1	1	0.498046634
C2H2	scaffold17894	AT1G72416.3	0.984662784	0.515236808
bZIP	C352249	AT1G61800.1	0.976846858	0.409009483
SET	C347463	AT1G08650.1	0.953023357	0.454410328
WRKY	C344833	AT2G30040.1	0.93985445	0.493192349
**TF Family**	**TSIDs**	**ATID**	**K_PB_Yellow**	**GS_PB_Yellow**
**Yellow Module (Pusa Bold)**				
bZIP	scaffold33254	AT2G23350.1	0.991272394	0.401528471
C2H2	C353855	AT4G25340.1	0.987034074	0.478533088
bZIP	scaffold500	AT1G49760.2	0.986043424	0.514279776
bZIP	scaffold501	AT1G49760.2	0.982596836	0.528334689
C3H	C350299	AT3G53460.3	0.981115832	0.429174095
**TF Family**	**TSIDs**	**ATID**	**K_PJK_Blue**	**GS_PJK_Blue**
**Blue Module (Pusa Jaikisan)**				
FAR1	scaffold43285	AT2G14170	0.99388054	0.449199376
C3H	scaffold10084	AT5G66760	0.99406313	0.443595033
NAC	scaffold45345	AT3G44110	0.990036914	0.455946169
SNF2	scaffold4567	AT5G60670	0.988754	0.415705034
FHA	C350767	AT1G53750	0.986395	0.472080454

Pusa Bold and Pusa Jaikisan. Each module with top-5 TFs, their transcript sequence ID (TSID), ATID (Arabidopsis TAIR ID names), K (Scaled connectivity), GS (Gene significance) are shown in table.

### Conserve module identification in both the cultivars in response to nitrate treatment

To identify the conserved module in response to nitrate treatment, the significant modules of PB were compared to the corresponding modules of PJK (Supplementary Fig. S10). The yellow module of PB, was found to be the most conserved module with maximum Zsummary (64.23) (Supplementary Table S10), and 483 genes out of 622 were conserved against corresponding brown module in PJK. Since the modules (Yellow and brown) are conserved in both datasets which implies that these modules may represent the signature pathway under nitrate response. We have also identified the shared HUB TF genes among these modules (Supplementary Table S11), which were found to have similar scaled connectivity (K) indicating importance of these genes in both datasets under nitrate response, thereby considered as signature genes (Supplementary Table S11).

## Discussion

Plant nitrogen use efficiency (NUE) is an important factor in determining crop growth and yield. High application of N-fertilizers is considered to be the costliest input in terms of financial and environmental losses^25^. Thus, improving plant NUE is an important area of research. In order to improve plant NUE, it is important to understand the crop nitrogen response at both physiological and molecular levels in response to external nitrate supply. Transcriptomic approach, employed to study N-response in several plants have contributed to understand plant response to exogenous change in nitrogen status at molecular level^3,26–28^. Moreover, comparative transcriptomic studies on high and low N-use efficient cultivars of several crops^10,11,13^ have also contributed to understand the complexity of crop N-response. Several novel transcripts have been identified from these studies, which may play important role in improving plant NUE. The present study was aimed to understand nitrate response in two cultivars of *B*. *juncea* using transcriptome and weighted gene co-expression network analyses.

Nitrogen is an important constituent for plant growth and development. Several processes in plants, like macromolecule (DNA, RNA and protein) biosynthesis, photosynthesis that ultimately affect plant growth and yield depend on the adequate supply of nitrogen^29^. It has been observed that high N-use efficient cultivars produce higher plant biomass as compared to the low N-use efficient cultivars^30^. In the present study, PB was found to exhibit higher root and shoot biomass as compared to PJK with increasing external nitrate concentration. This supports the fact that PB is more N-use efficient cultivar as compared to PJK. The reduction of nitrate to nitrite by nitrate reductase (NR) is considered to be the first rate limiting step for plant N assimilation^31^. The NR activity (NRA) in both the cultivars was found to be induced with increasing nitrate concentration, which indicates that nitrate has positive effect in inducing NRA in *B*. *juncea*, unlike *B*. *napus* seedlings, where ammonium supply in absence of nitrate was found to induce NRA more than nitrate^23^. The induction of NRA by nitrate has been already reported in maize^32^ and barley^33^. Moreover, genotypic difference in NRA has been reported in Chinese cabbage^34^. The NRA of PB was found to be significantly higher as compared to PJK in all treatments indicating that the PB exhibit higher capacity to reduce nitrate into nitrite and finally to ammonium, which is incorporated into amino acids. These observations clearly indicate that PB performs better in terms of growth and physiological parameters as compared to PJK in response to nitrate treatment.

### Early nitrate treatment induces distinct transcriptional machinery in both the cultivars

In this study, we adopted RNA-seq approach to understand the molecular response of both the cultivars of *B*. *juncea*, PB and PJK in response to various nitrate treatments. The transcriptional changes in response to early nitrate treatment include several transcription factors and protein kinases that may have important role in nitrate mediated signaling. Our transcriptome study suggested that there was a distinct transcriptional response in both the cultivars in response to early nitrate treatment that might serve as an important factor governing nitrogen use efficiency of *B*. *juncea*.

### Involvement of TF families and protein kinases in nitrate mediated signaling response

As a signaling molecule, nitrate can regulate expression of several genes. A number of transcription factors and protein kinases are known to be involved in nitrate mediated signaling^10,35^, which in turn regulate several nitrate responsive genes. Several TFs like, MYB, WRKY, NAC, bHLH, bZIP, AP2-EREBP, GATA and DOF have already been reported to be involved in plant nitrogen regulatory network^10,21–23^. Comparative analysis of various transcription factors that are found to be induced in both the cultivars in response to an early nitrate treatment (20 min) revealed that MYB TFs in PB and AP2/EREBP TFs in PJK were most abundant TF families. Previously, the involvement of MYB TF in controlling the expression of nitrate transporters and assimilatory genes has been reported^21,36^. Although, the exact role of AP2/EREBP TF in nitrate mediated signaling is not known, but involvement of this TF in response to nitrogen deficiency has been observed in rice^37^. Overall, the differential expression of various transcription factors in response to an early nitrate treatment in both the cultivars may be one of the factors responsible for differential NUE of these cultivars.

**Figure 7:**
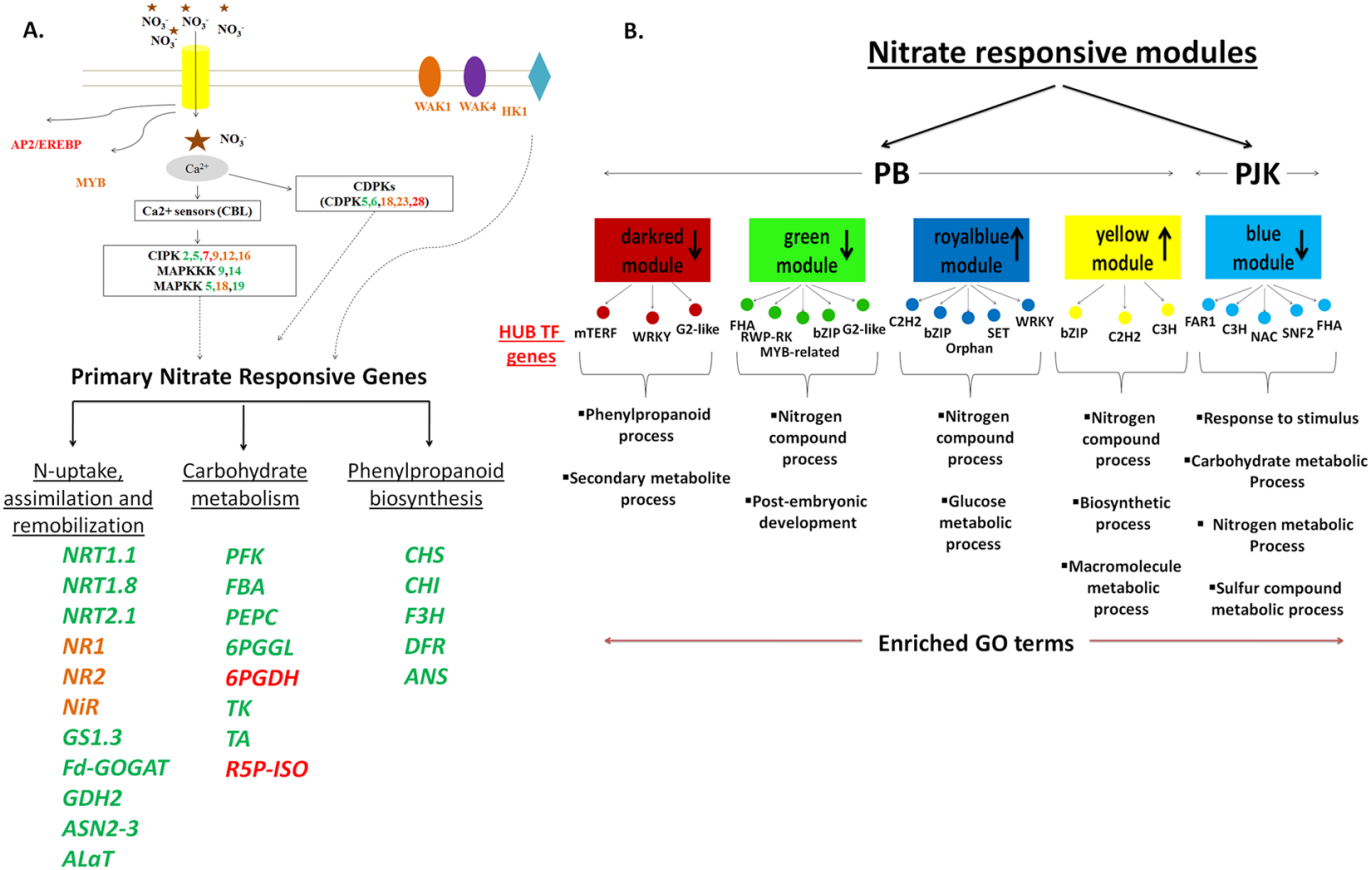
Proposed model depicting nitrate responsive genes and nitrate regulated network in *B*. *juncea* cv. PB and PJK: Nitrate as a signal leads to activation of several signalling pathways which include transcription factors (MYB, AP2/EREBP) and protein kinases (WAK, HK, CIPK, CDPK) (**A**). These downstream regulatory component lead to the transcriptional regulation of several pathways involved in plant growth and development. The nitrate responsive genes induced early in high NUE cultivar, PB or low NUE, cultivar PJK or in both are represented by green, red and orange color, respectively (**A**). Co-expression network analysis revealed four significantly enriched nitrate regulated modules in PB and one in PJK (**B**). The enriched GO terms associated to each module are also shown. The black arrows inside the respective nitrate-regulated modules represent up or down regulation in response to nitrate treatment (**B**). WAK: wall associated kinases, CBL: Calcineurin B- like protein, CIPK: CBL-interacting protein kinases, CDPK: Ca^2+^ dependent protein kinases, MAPKKK: Mitogen activated protein kinase kinase kinase, MAPK: Mitogen activated protein kinase, NRT: Nitrate transporters, NR: Nitrate reductase, NiR: Nitrite reductase, GS: Glutamine synthetase, Fd-GOGAT: Glutamate synthase, GDH2: Glutamate dehydrogenase, ASN: Asparagine synthetase, ALaT: Alanine aminotransferase, PFK: Phosphofructokinase, FBA: Fructose-bisphosphate aldolase, PEPC: Phosphoenolpyruvate carboxylase, 6PGGL: 6-phosphogluconolactonase, 6PGDH: 6-phosphogluconate dehydrogenase, R5P-ISO: Ribose 5-phosphate isomerase A, TK: Transketolase, TA: Transaldolase, CHS: Chalcone synthase, CHI: Chalcone-flavanone isomerase, F3H: Flavanone 3-hydroxylase, DFR: Dihydroflavonol 4-reductase, ANS: Anthocyanidin synthase.

Protein Kinases are also known to play important roles in nitrate signaling^38^. In our transcriptome data, histidine kinase 1 (HK1) was found to be up regulated after 20 min of nitrate supply in both the cultivars. Role of histidine kinases (HKs) has been reported in variety of plant responses such as under salinity and drought^39,40^. Moreover, the involvement of HK in nitrate signaling was reported in *E*. *coli*^41^, where two HKs, Nar X and Nar Q bind to nitrate and nitrite, respectively via their P-box and phosphorylate response regulators such as Nar L and Nar P, which in turn regulate expression of downstream genes. Therefore, early up regulation of HK1 in both the cultivars in response to low nitrate treatment may reveal its involvement in nitrate mediated signaling in *B*. *juncea*. Additionally, two wall associated kinases (WAK1 and WAK4) were also found to be induced in response to an early nitrate treatment suggesting their role in nitrate signaling. In Arabidopsis, WAK1 was found to be involved in response to aluminium^42^ and tolerance against pathogen^43^, but its role in nutrient response is not yet known. However, the reduction in expression of *WAK1* gene was reported under high nitrate condition in *Arabidopsis*^4^. Role of WAK4 in root mineral nutrition has been studied in *Arabidopsis*, where its expression was found to be induced by Na^+^, K^+^, Cu^+^, Zn^+^ and Ni^+^ ions^44^. A link between calcium and nitrate signaling has been well studied in maize and *Arabidopsis*^38,45^. In plants, regulation of nitrate sensing and uptake by calcium signaling requires Ca^2+^ dependent protein kinases (CDPKs) and calcineurin B- like protein/ CBL-interacting protein kinases (CBL/CIPKs). In our transcriptome data, several CDPKs and CIPKs were differentially induced in response to an early nitrate treatment in both the cultivars (Fig. 7). The CDPK family is thought to be involved in majority of Ca^2+^ mediated downstream signaling events^46^. Five CDPKs (CDPK 5, 6, 18, 23 and 28) were found to be induced in response to early nitrate treatment. The CDPK18 and CDPK23 were commonly induced in both the cultivars, whereas CDPK5-6 and CDPK28 were specific to PB and PJK, respectively. In Arabidopsis, CDPK23 was reported to be involved in activation of anion channels like SLAC1^47^, hence its early induction in both the cultivars reflect its role in nitrate sensing and uptake. In our study, six CIPK genes (CIPK 2, 5, 7, 9, 12 and 16) were induced early in response to nitrate treatment, three (CIPK 9, 12 and 16) of which were commonly induced in both the cultivars, whereas CIPK 2, 5 and CIPK9 were specific to PB and PJK, respectively. Previous study has revealed the involvement of two protein kinases, CIPK8 and CIPK23 in regulating nitrate-mediated response in plants^38^. Therefore, we hypothesize that these identified CIPKs might be important component of nitrate signaling in *B*. *juncea*.

### Interaction between nitrate and cytokinin mediated signalling

Nitrate signal can be transferred through two-component system mediated by cytokinin. Cytokinin level in plants also provides indication of their nitrogen status^45^. Moreover, both nitrate and cytokinin function as root to shoot long distance signal of nitrogen supplementation. It has been also observed that genes of nitrogen metabolism are activated by cytokinin^48^. Moreover, *IPT3* gene was found to be nitrate inducible in *Arabidopsis*^49^. This close interaction between cytokinin and nitrogen metabolism pathway instigated us to investigate the cross-talk of nitrate and cytokinin signaling in *B*. *juncea*. Several genes involved in cytokinin signaling were found to be induced at 2 h of high nitrate (4 mM KNO_3_) treatment in our transcriptome data. For e.g. A CRE1 (cytokinin response 1; scaffold 41778), which is one of the best characterized cytokinin His kinase receptors was found to be induced at 2 h of high nitrate supply. One of the primary cytokinin responsive genes, AAR5 (C125339) induced at 2 h of high nitrate treatment in our transcriptome data was also reported to respond towards changing nitrogen level in *Arabidopsis*^50^. The induction of these genes in response to nitrate treatment might suggest that some of the non-specific responses of nitrate could be triggered by cytokinin in *B*. *juncea* as reported in *Arabidopsis* and maize^51^ and CRE1 and ARR5 may in turn act as common elements in nitrate and cytokinin mediated signaling in *B*. *juncea*.

### Rapid induction of nitrate transporters and assimilatory genes in PB in response to nitrate treatment

Our RNA-seq results indicate the differential expression of nitrate uptake and assimilatory genes in both the cultivars of *B*. *juncea* that might be one of the factors contributing to their differential behavior in response to nitrate treatment. The NRT1.1 transporter is one of the most important low affinity nitrate transporters^52^. It also acts as a nitrate sensor and was found to be involved in nitrate signaling^53^. Early induction of *NRT1.1* in PB may in turn contribute to rapid uptake of nitrate as compared to PJK. Induction of *NRT1*.*8* gene that was previously reported to be involved in long distance transport of nitrate to the shoot was also observed in our transcriptome data^54^. The increase in expression of nitrate reductase (NR) genes in response to nitrate treatment mainly in PB might suggest its better ability to reduce nitrate. Previous microarray studies have ranked *NRT1*.*1* and *NR* genes as top most nitrate inducible genes^3,27^. Increase in expression of ammonia assimilatory proteins has been well reported in PB as compared to PJK under low N-conditions^20^. In the present study, expression of several transcripts encoding enzymes like glutamine synthetase, glutamate synthase and glutamate dehydrogenase were found to be induced early in PB. Comparative transcriptome studies have also suggested increased expression of nitrogen metabolism genes mainly in high NUE genotype^11^. Alanine aminotransferase (ALaT) is another important assimilatory enzyme that catalyzes the synthesis of alanine and 2-oxoglutarate from pyruvate and glutamate vice-versa. Thus, ALaT is an important enzyme that maintains the carbon and nitrogen metabolism in plant system and might play a significant role in maintaining the carbon and nitrogen balance in plant system^55^. Moreover, overexpression of this enzyme in some crops, like rice and barley has increased plant biomass, seed yield and NUE when grown under low nitrate supply^56,57^. The early induction of ALaT gene in PB in response to nitrate treatment might be a strategy to adjust the nitrogen and carbon shuttle. Overall, these results suggested that, early induction of N-assimilatory genes in PB may be one of the main factors contributing to its high NUE (Fig. 7A).

### Enrichment of anthocyanin pathway at low-N condition

Accumulation of anthocyanin was found to be an adaptive response to acclimatize under N- limiting condition in *Arabidopsis*^58^. The biosynthesis of anthocyanin takes place through phenylpropanoid pathway. Under nitrogen limiting condition, the phenylpropanoid pathway is fluxed into anthocyanin production, which in turn helps plant to adapt under low N-conditions^58^. Elevated expression of several genes of phenypropanoid pathway that lead to anthocyanin production was observed in cucumber seedlings in response to nitrogen deficiency^7^. In our transcriptome data, enrichment of phenypropanoid pathway under low nitrate treatment was observed in PB. Moreover, several genes involved in anthocyanin production *viz*. chalcone synthase (CS; scaffold44688), anthocyanidine synthase (ANS; scaffold45666), chalchone flavanone isomerase (CHI; C327197), dihydroflavonol 4-reductase (DFR, scaffold360) and flavanone 3-hydoxylase (F3H; scaffold26794) were also found to be induced in response to low nitrate condition in PB (Fig. 7A). Enrichment of phenypropanoid pathway in presence of low nitrogen condition might be an adaptation strategy of *B*. *juncea* to survive under nitrate limiting conditions.

### Co-expression network analysis reveals important nitrate regulatory modules

Our co-expression network analysis has identified nitrate regulatory modules (4 modules in PB and 1 module in PJK) that were found to be significantly up- or down-regulated in response to nitrate treatment. The criteria for selecting the significant modules have been previously used by several studies^59,60^. The nitrate regulatory network in plants is highly complex and coordinated with several other processes in plants^61^. Transcription factors are known as important regulatory proteins that can regulate expression of several genes, simultaneously^62^. Therefore, we have identified several HUB TF genes in each significant module that might interact with the other genes involved in specific pathway. As HUB genes play important role in gene network^63^, we have identified HUB TF genes with maximum number of connection in nitrate regulated modules. The top-5 HUB TF genes were further selected in each module based on the scaled connectivity (K) and gene significance (GS). In the present study, the mTERF TFs were ranked as top-HUB genes in darkred module of PB. The ortholog of mTERF encoding gene in *Arabidopsis* (AT1G03940) has been reported to be involved in phenylpropanoid pathway^64^. As we have stated earlier that enrichment of phenylpropanoid pathway in PB under low nitrate condition could be an adaptation strategy, however several reports have also suggested that sufficient nitrate condition leads to repression of genes of phenylpropanoid pathway^4,65^. Our network analysis suggests that mTERF TF could be involved in downregulating phenypropanoid pathway in response to nitrate treatment. The green module contains top GO terms like nitrogen compound metabolic process and post-embryonic development process. The HUB gene from green module, RWP-RK has been shown to play important role in primary nitrate response by acting as a key player downstream to nitrate signaling leading to induction of several nitrate responsive genes^66^. Therefore, based on the network analyses, it appears that nitrogen processes in green module could be regulated by RWP-RK HUB TF genes. The enriched terms in royalblue module were found to be associated with nitrogen and carbon metabolic processes, which are known to be highly coordinated^27,67^. The top HUB TF gene in this module belongs to Orphan TF family. The ortholog of this gene in *Arabidopsis* (AT1G78050) has been known to be induced by nitrate^3,68^. Another HUB TF gene in this module, SET, encodes a phosphoenolpyruvate carboxylase kinase 1 that has been reported to be an important enzyme for coordination of carbon and nitrogen metabolism^69^. The yellow module was found to be enriched with several GO terms that were reported to be upregulated under nitrate response^23^. The *Arabidopsis* ortholog (AT2G23350) of bZIP (scaffold33254), which was also found to be significantly enriched in yellow module, is known to be involved in protein synthesis^70^. The blue module of PJK that was found to be downregulated in response to nitrate treatment was enriched with terms associated with protein synthesis that was generally upregulated under nitrate response^23^. Moreover, several other terms, like hexose metabolic process, nitrogen compound metabolic process, cellular component biogenesis, ribonucleo protein complex biogenesis and ribosome biogenesis were also enriched in this module. The gene encoding nitrate reductase (NR) enzyme (C342425) was also found in this module. Since protein synthesis and carbohydrate metabolism are crucial for the nitrate reductase activity^71^, the down-regulation of such terms in PJK may correspond to the reduced activity of NR towards nitrate response. Therefore, the HUB genes identified through network analysis might suggest their importance in regulating nitrate mediated signaling, their interaction with carbohydrate pathway and also the differential behavior of both the cultivars in response to nitrate treatment.

## Conclusions

In the present study, we have performed the transcriptome analysis of two cultivars of *B*. *juncea* cv. PB and PJK in response to various nitrate treatments. Our RNA-seq analysis provides useful information for understanding the nitrate-mediated molecular mechanism in both the cultivars. The transcriptome data clearly shows that both the cultivars adapt different strategies at molecular level in response to different nitrate conditions. Moreover, the significant differential induction of 28 genes involved in N-transport (NRT, AMT), assimilation (NR, NiR, GS, GOGAT) and remobilization (GDH, AS, ALaT) in two cultivars in response to nitrate has major influence on different nitrogen uptake and utilization efficiency of these cultivars. In addition, the co-expression network analysis of 25,239 common genes obtained from the RNA-seq data of two cultivars has revealed several nitrate regulatory modules that might be associated with the regulation of phenypropanoid biosynthesis, carbon: nitrogen interaction and nitrate reductase activity in *B*. *juncea*. Moreover, the identified hub genes in these modules might also suggest their involvement as key regulators in nitrate regulatory network of *B*. *juncea*. In future, it would be interesting to functionally characterize the nitrate responsive transcripts, especially from high NUE cultivar, which may be the potential candidates to improve NUE of *B*. *juncea* and related crops.

## Methods

### Plant materials and growth conditions

Healthy seeds of *Brassica juncea* cv. PB and PJK were surface sterilized by immersion in 70% ethanol for 2–3 minutes and rinsed with autoclaved distilled water, 3–4 times. Seeds were spread on moist filter paper in petriplates and kept in dark at 22 ± 2 °C for 3d stratification and then transferred to light. One week old seedlings were transplanted in 9 cm diameter pots containing vermiculite and peat moss (3:1) for semi-hydroponics culture. The seedlings were supplied with Hoagland nutrient solution: 1 mM KH_2_PO_4_, 0.5 mM MgSO_4_, 0.25 mM CaSO_4_, 20 µM Fe-EDTA, 25 µM H_3_BO_3_, 2 µM ZnSO_4_, 2 µM MnSO_4_, 0.5 µM CuSO_4_, 0.5 µM (NH_4_)_6_Mo_7_O_24_ (pH 6.5). The ammonium succinate (2.5 mM) was added in the nutrient solution as a sole source of nitrogen until the emergence of true leaves. After emergence of true leaves (11^th^ day after transplanting in pots) the plants were treated with three different concentrations of nitrate; low nitrate (0.25 mM KNO_3_), medium nitrate (2 mM KNO_3_) and high nitrate (4 mM KNO_3_) in Hoagland solution. Seedlings treated with equal amount of KCl in place of KNO_3_ were treated as control (Supplementary Fig. S11). Seedlings were grown in growth chamber at 22 ± 2 °C with 16 h/8 h photoperiod, 150 μmol m^−2^ s^−1^ light intensity and 50% relative humidity. The whole seedlings were harvested after 20 min, 2 h, 12 h, 24 h, 3 d and 7 d of treatments. For transcriptome analysis, the collected samples were immediately frozen in liquid nitrogen and stored at −80 °C for future use. The growth parameters of both the cultivars were recorded at 21 d after giving various nitrate treatments. For estimation of nitrate reductase activity (NRA) and nitrate content, seedlings were freshly harvested at 2 h, 24 h, 3 d, 7 d and 15 d after treatments and used for analyses.

### Growth parameters, NR activity and nitrate content estimation

Various growth parameters like, root length, shoot length, root and shoot fresh weight and dry weight were recorded in both the cultivars at respective time points. The NR activity was determined by method described^72^. The nitrate content was estimated as described^73^.

### Tissue sampling, RNA isolation, cDNA library preparation and Illumina sequencing

In total, 42 samples comprising of 21 samples from each cultivar were used for transcriptome analysis (Supplementary Table S1). Total RNA was extracted from whole seedlings (pooled from three biological replicates) as described^74^, concentration and quality of RNA were checked using NanoDrop 1000 (NanoDrop Technologies, USA) and Bioanalyzer (Agilent technologies, USA), respectively and 5 µg total RNA of each sample with RNA integrity number ≥7 was used for library preparation using TruSeq RNA sample preparation kit v2 (Illumina Inc., USA). Each sample was tagged with unique TruSeq index tag to prepare multiplexed libraries. The quantification of prepared libraries was performed on Qubit fluorometer using Qubit dsDNA BR assay kit (Life Technologies, USA). The insert size and purity of libraries were further checked on Bioanalyzer chip DNA 1200 series II (Agilent Technologies, USA). All the 42 libraries were divided in to seven pools, each containing six libraries, and each pool was loaded in each of the seven lanes of flow cell for cluster generation in cluster station using TruSeq PE cluster generation kit v5 (Illumina Inc., USA). The amplified clusters were then used to perform paired-end (PE) (2 × 72) sequencing on Genome Analyzer IIx (Illumina Inc., USA). The raw reads generated from Illumina GAIIx were submitted as BioProject PRJNA383771 to the NCBI Sequence read archive.

### *De novo* assembly and sequence clustering

Paired-end (PE) read sequences of length 72 bp each with an insert-length of 260 bp were generated using CASAVA package. Quality assessment of read sequences was performed using read quality filtering tool, filteR in which poor quality reads and adapter contaminated reads were filtered-out. *De novo* assembly of good quality reads was performed using assembler SOAPdenovo-trans (http://soap.genomics.org.cn) which was run on different k-mer sizes ranging from 19–71 based on good quality read sequences and corresponding contigs/scaffolds were produced for each k-mer. Tool GapCloser was used to close the gaps emerged during the scaffolding process. CD-HIT-EST version 4.6, a clustering program was used to search similar sequences with minimum similarity cut-off of 90% (http://weizhongli-lab.org/cd-hit). Another clustering step was performed using TGICL-CAP3 version 2.2.26 program at 90% identity to cluster the similar sequences (http://www.tigr.org/tdb/tgi/software).

### Mis-assembly prediction and assembly validation

In order to detect the mis-assembled sequences, sequence similarity with closely related organism, *Brassica rapa* (http://ebi.edu.au/ftp/software/software/ensembl/eg-dumps/eg-13/plants/) was carried out. Tophat and Cufflinks were used to assemble reference sequences using good quality read^75^. To determine the sequence similarity among the reference and *de novo* assembled sequences, BLASTN analysis was performed considering reference sequences as database and *de novo* assembled sequences as query with an E-value threshold of 1e-05. To estimate the credibility of correctly assembled sequences, a total of 5,518 experimentally validated EST sequences of *Brassica juncea* available at NCBI were used. These EST sequences were scanned against the correctly assembled transcripts using BLASTN with an E-value threshold of 10^−5^.

### Homology search, unigene identification and Sequence annotation

The correctly assembled transcripts were searched against NCBI NR protein sequence database using BLASTX at an E-value threshold of 10^−5^. The transcripts that had no sequence similarity but may belong to different regions of similar gene were identified and clustered using Dissimilar Sequence (DS) clustering approach. Annot8r program was used to annotate the correctly assembled transcripts with an E-value threshold of 10^−1^ (http://www.nematodes.org/bioinformatics/annot8r). To derive transcription factor family related information PlnTFDB database was used (http://planttfdb.cbi.pku.edu.cn). All the unigenes were searched against this database using BLASTX with an E-value threshold of 10^−5^.

### FPKM calculation, differential expression measurement and gene enrichment analysis

To measure the expression of assembled transcripts, FPKM values were determined using RNA-seq expression estimation by Expectation-Maximization (RSEM; https://deweylab.github.io/RSEM). Fold change was calculated and transcripts that exhibited two fold or above differential expression were considered as differentially expressed. AgriGO’s Singular Enrichment Analysis (SEA) module was used to identify the enriched Gene Ontology terms (http://bioinfo.cau.edu.cn/agriGO/analysis.php). The enrichment analysis was performed at significance level of 0.05. The available GO terms of all the unigenes were taken as background whereas, the query list contained only those GO terms which are differentially expressed in that particular comparative condition. Hyper-geometric statistical test and Bonferroni correction methods were applied.

### Reference based assembly of transcriptome data with published genome of *B*. *juncea*

The *de novo* assembled transcripts were compared with the genes from recently published genome^24^. For that purpose, trancriptome reads were mapped across the reference genome with annotated GTF file using Bowtie. FPKM values were determined using RSEM for all the genes. To find the closest genes for all the transcripts, BLASTN was used. Minimum 100 Bit-score and E-value < 1-E05 were used as the cutoff values to look for best representative gene for each trancript. Differential expression for all these representative genes was calculated using log fold change. Overlap between genes and transcripts was found on the basis of differential expression patterns. The entire analysis with reference genome was performed exactly on the similar line as was done for the *de novo* assembly based study. This ensured technical consistency in the analysis.

### Validation of RNA-Seq data by qRT-PCR

For quantitative real-time PCR (qRT-PCR) analysis, 5 µg of total RNA was reverse transcribed using Revert-aid H Minus Reverse Transcriptase kit (Thermo Scientific, USA) in a final volume of 20 µL according to the manufacturer’s instructions. The qRT-PCR reaction was performed on StepOne plus real-time PCR machine (Applied Biosystems, USA). Each reaction contained 2.5 µL diluted cDNA, 10 mM each of forward and reverse gene specific primers and 5 µL SYBR Green qPCR Master Mix (Applied Biosystems, USA) in a final volume of 10 µL. The qRT-PCR reaction was performed with three technical and three biological replicates. The thermal cycling program used was as follows: 4 min at 94 °C, 40 times cycling for 30 s at 94 °C, 30 s at annealing temperature and 72 °C for 30 s. The specificity of reactions was checked by melt curve analysis (Supplementary Fig. S12). The sequence information of primers and CT values of the qRT-PCR results of all the genes for all the samples are shown as Supplementary Tables S12 and S13, respectively. The *BjUbq9* was used as an internal reference^76^. The relative expression ratio of each gene was calculated using 2^−ΔΔCT^ method as described^77^.

### Weighted gene co-expression network analysis

Normalized RNA-seq datasets of both, PB and PJK were analyzed through network biology approach to study their differential response under nitrate treatments. Genes with excessive missing values and sample outliers were removed from both datasets using Weighted Gene Co-Expression Network Analysis (WGCNA) library^78^ of R statistical package version 3.0.1 and common genes obtained were further utilized in construction of two separate weighted networks. Pearson correlation matrices corresponding to gene expression were computed for each weighted network and transformed into connection strength matrices using a power function that best suits its scale-free behavior^79^. Connection strengths were transformed into a topological overlap similarity (TOM) measure which was further used to compute dissimilarity TOM (DistTOM)^78^. DistTOM similarity measure between two genes ($i$ and $j$) is described as:

$$TO{M}_{ij}=\frac{{l}_{ij}+{a}_{ij}}{min({k}_{i},{k}_{j})+1-{a}_{ij}}$$

$$DisTO{M}_{ij}=1-TO{M}_{ij}$$

where, ${l}_{ij}=\sum _{u}{a}_{iu}{a}_{uj}$, and ${k}_{i}=\sum _{u}{a}_{iu}$ is the node connectivity and ${a}_{{ij}}$ is the network adjacency. Finally, DistTOM measure along with average linkage hierarchical clustering was implemented in DynamicTree Cut algorithm^80^ to obtain comparable modules from both datasets with appropriate value of deepsplit. Data under various nitrate treatments and time points was integrated with expression data to identify modules significantly associated with nitrate response. Modules with correlation (r ≥ 0.4) and p-value (≤0.05) were extracted for further inquisition. Gene ontology (GO) for biological process and Kyoto Encyclopedia of Genes and Genomes (KEGG) pathway annotation of the significant modules were performed using the Database for Annotation, Visualization and Integrated Discovery (DAVID) *v*6.7 (https://david.ncifcrf.gov). Enrichment analysis was carried out with Singular Enrichment Analysis (SEA) of agriGO web based tool (http://bioinfo.cau.edu.cn/agriGO/analysis.php). Hypergeometric test with Bonferroni correction was applied for selecting these statistically significant terms. Module identifiers in the PB were compared to match the most similar module in the PJK network based on significant overlap genes using overlap table function of WGCNA. To determine transcription factors (TFs) as central genes (hub genes) from significant modules their scaled connectivity (K) and genes significance (GS) was computed. VisANT tool (http://visant.bu.edu) was used to visualize differentially connected genes for various modules. The function module Preservation was used to compute module preservation, in terms of Z_summary,_ by comparing test network (PJK) against reference network (PB) i.e. module definitions from a reference network were taken and applied to a test network.

### Data availability

The Illumina sequence data from this study have been submitted as BioProject ID: PRJNA383771 to the NCBI Sequence read archive.

## Electronic supplementary material

Supplementary Figures

Supplementary Table S1

Supplementary Table S2

Supplementary Table S3

Supplementary Table S4

Supplementary Table S5

Supplementary Table S6

Supplementary Table S7

Supplementary Table S8

Supplementary Table S9

Supplementary Table S10

Supplementary Table S11

Supplementary Table S12

Supplementary Table S13
