# Supplementary material for: Transcriptome and Co-Expression Network Analyses Identify Key Genes Regulating Nitrogen Use Efficiency in Brassica juncea L
Source: Sci Rep. 2018 May 10;8:7451. doi: 10.1038/s41598-018-25826-6 (PMC5945678; doi:10.1038/s41598-018-25826-6)
Supplement: Supplementary file 1 — Supplementary Figures [file 41598_2018_25826_MOESM1_ESM.pdf]

# Transcriptome and Co-expression Network Analyses Identify Key Genes Regulating Nitrogen Use Efficiency in *Brassica juncea* L.

Parul Goel[1,2,#], Nitesh Kumar Sharma[1,2, #], Monika Bhuria[1,2], Vishal Sharma[1,2], Rohit Chauhan[1], Shivalika Pathania[1], Mohit Kumar Swarnkar[1], Vandna Chawla[1], Vishal Acharya[1], Ravi Shankar[1,2,*] Anil Kumar Singh[1,2,3,*]

[1]Department of Biotechnology, CSIR-Institute of Himalayan Bioresource Technology, Palampur-176061 (HP) India

[2]Academy of Scientific and Innovative Research, New Delhi

[3]ICAR-Indian Institute of Agricultural Biotechnology, PDU Campus, IINRG, Namkum, Ranchi-834010, India

[#] These authors contributed equally to this manuscript

*Correspondence and request for material should be addressed to A.K.S. (email: anils13@gmail.com) or R.S. (email: ravish@ihbt.res.in)

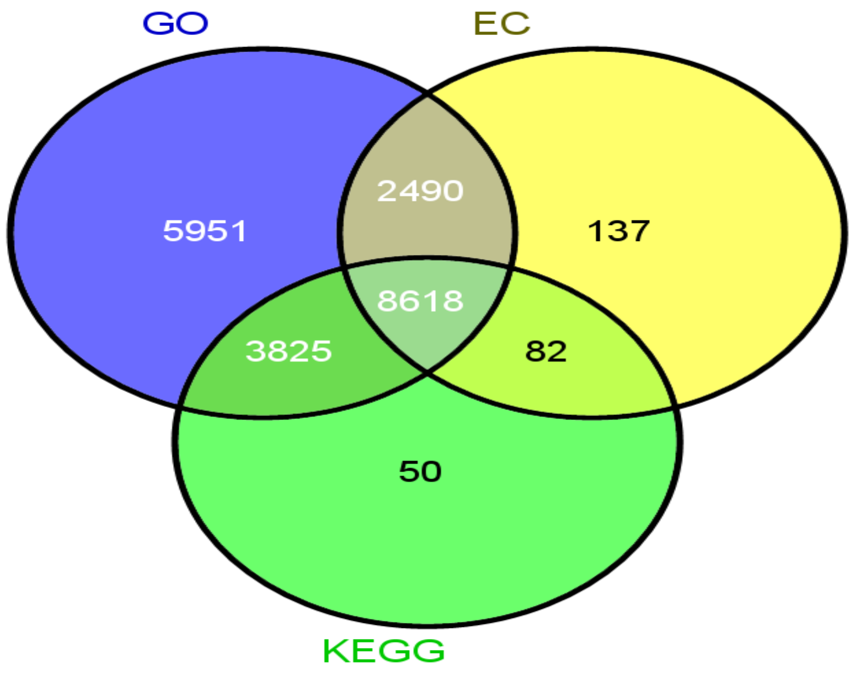


**Supplementary Fig S1:** Venn diagram showing transcripts annotated under GO, EC and KEGG

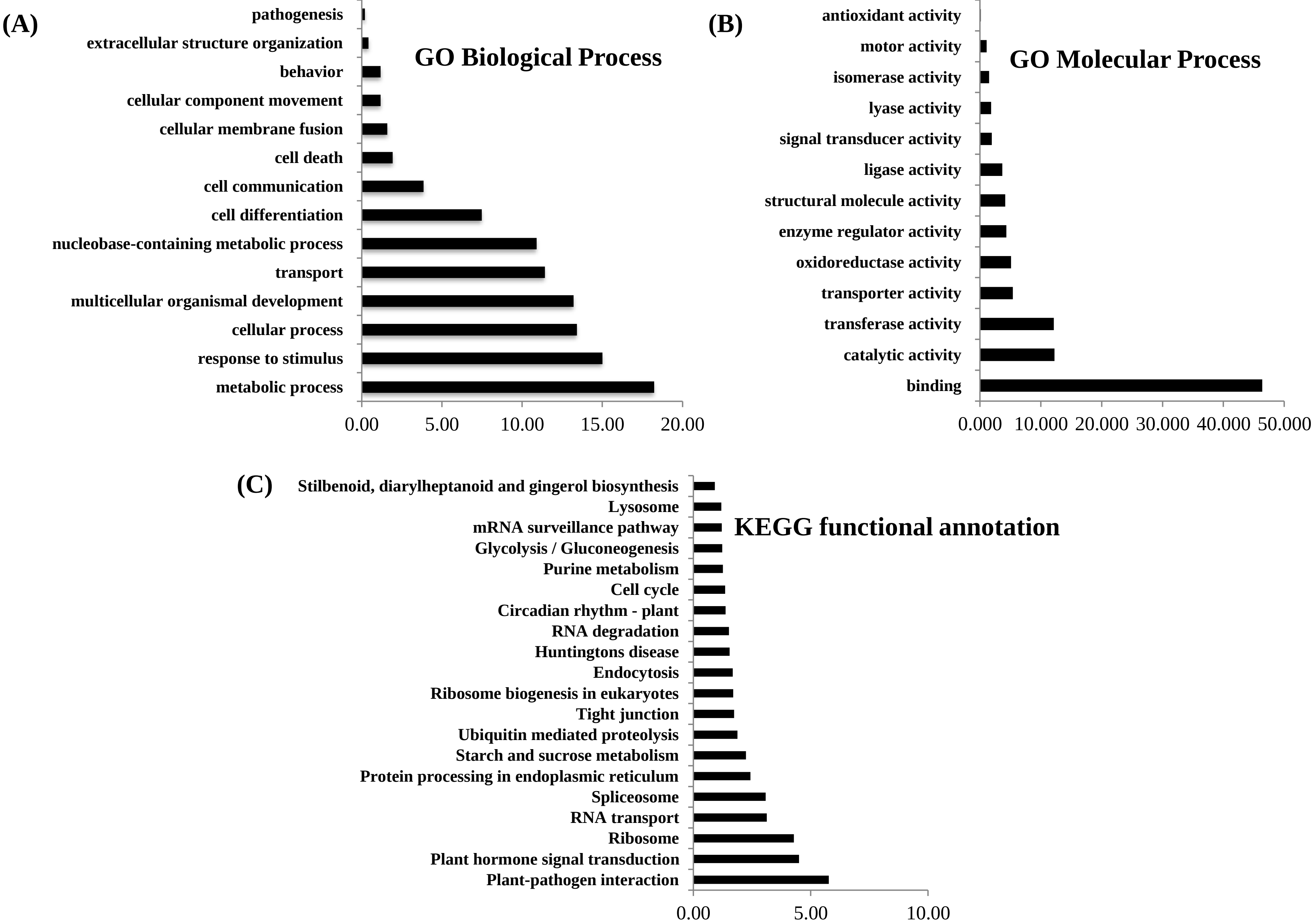


**Supplementary Fig S2: Functional Annotation detail of transcriptome data:** (A): Occurrence of different biological processes and molecular processes (B) based on GO slim categories with their corresponding percentage, (C): Top-20 KEGG pathways on the basis of their transcript abundance.

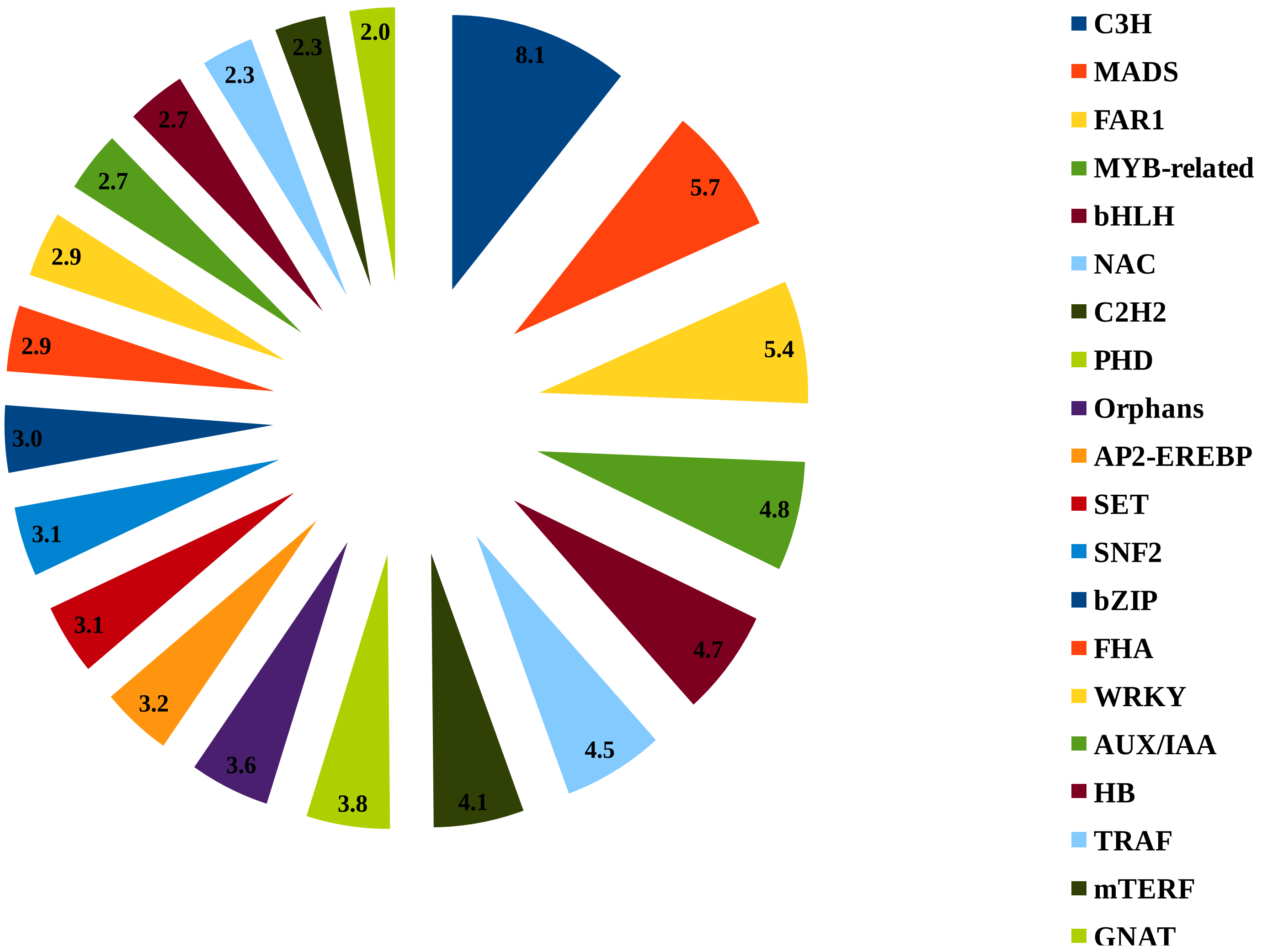


**Supplementary Fig S3: Top-20 transcription factor (TF) families on the basis of transcript abundance.**

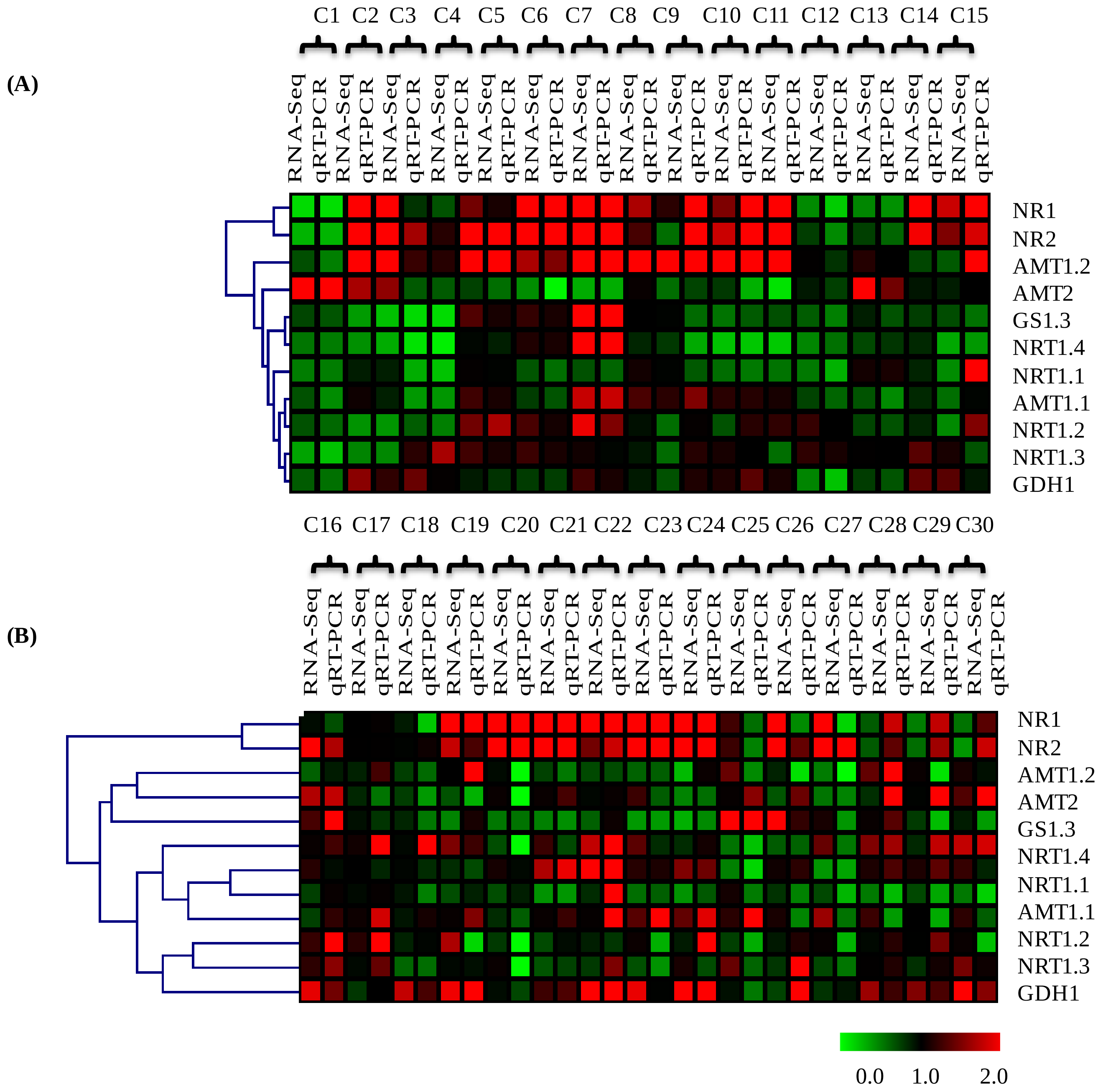


**Supplementary Fig S4:** Validation of the RNA-seq data from qRT-PCR: The expression level of eleven genes is measured through qRT-PCR in all 30 comparative conditions of (A) Pusa Bold and (B) Pusa Jaikisan. *Ubq9* was used as endogenous reference. The qRT-PCR data was significantly correlated with RNA-seq data ($r=0.76$, $p<0.0001$)

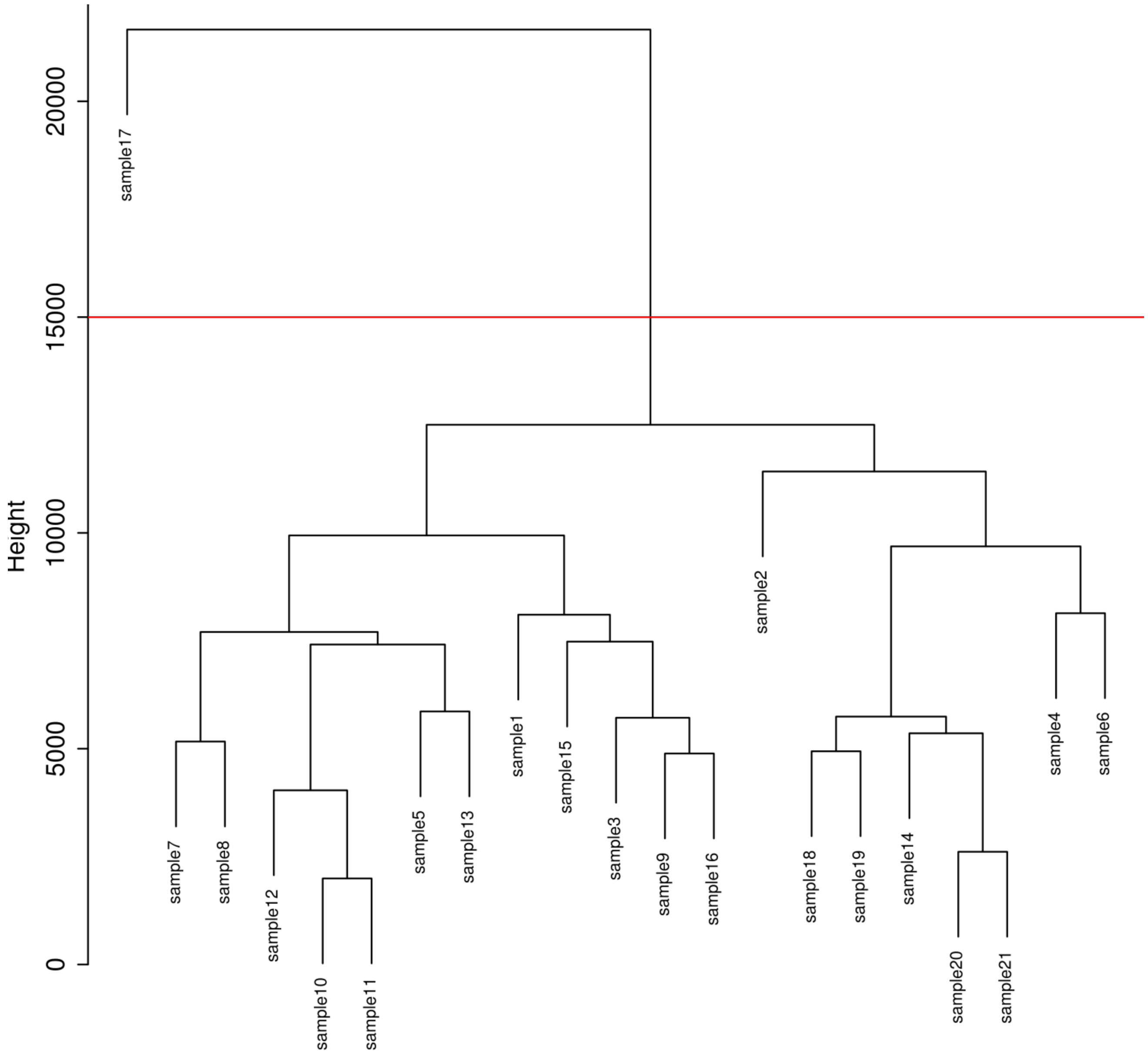


**Supplementary Fig S5: Filtering and pre-processing of data:** Sample 17 was eliminated as outlier from Pusa Bold.

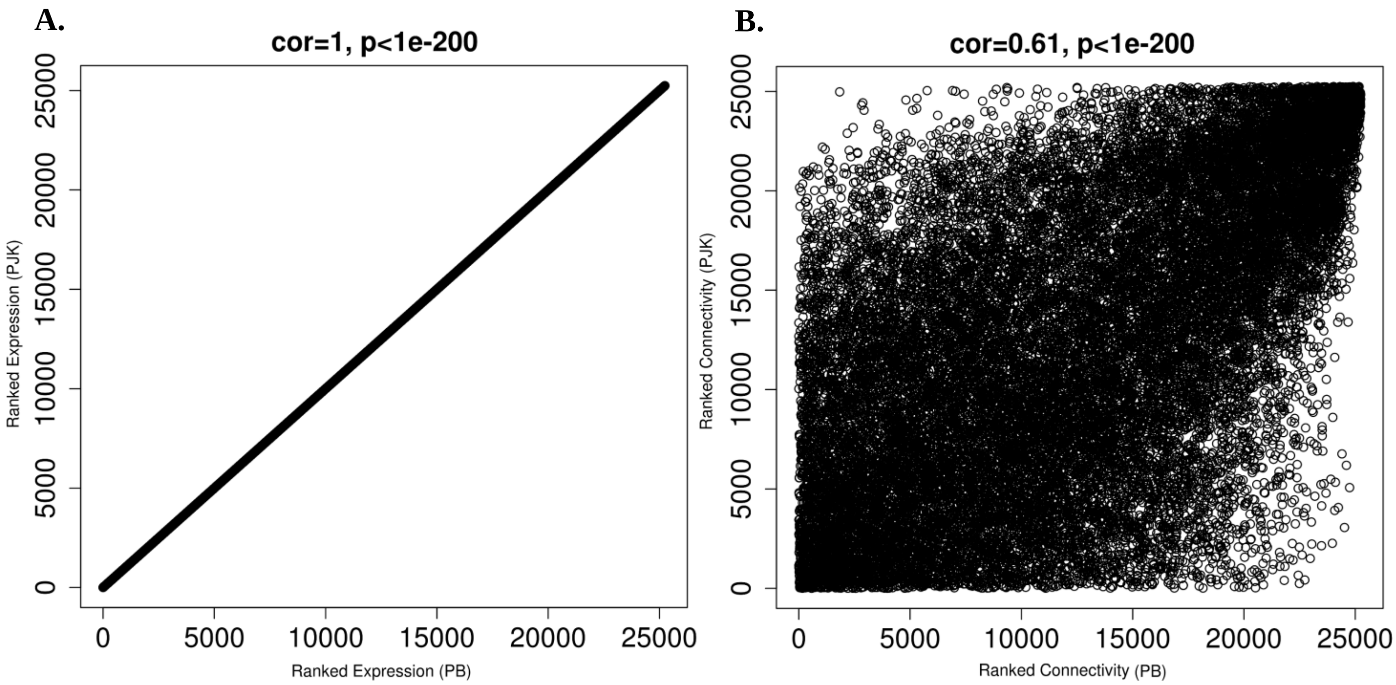


**Supplementary Fig S6:** Positive correlation and significant p-values of average gene expression (cor=1, p ≤ 1e-200) (a) and overall connectivity (cor=0.61, p ≤ 1e-200) (b).

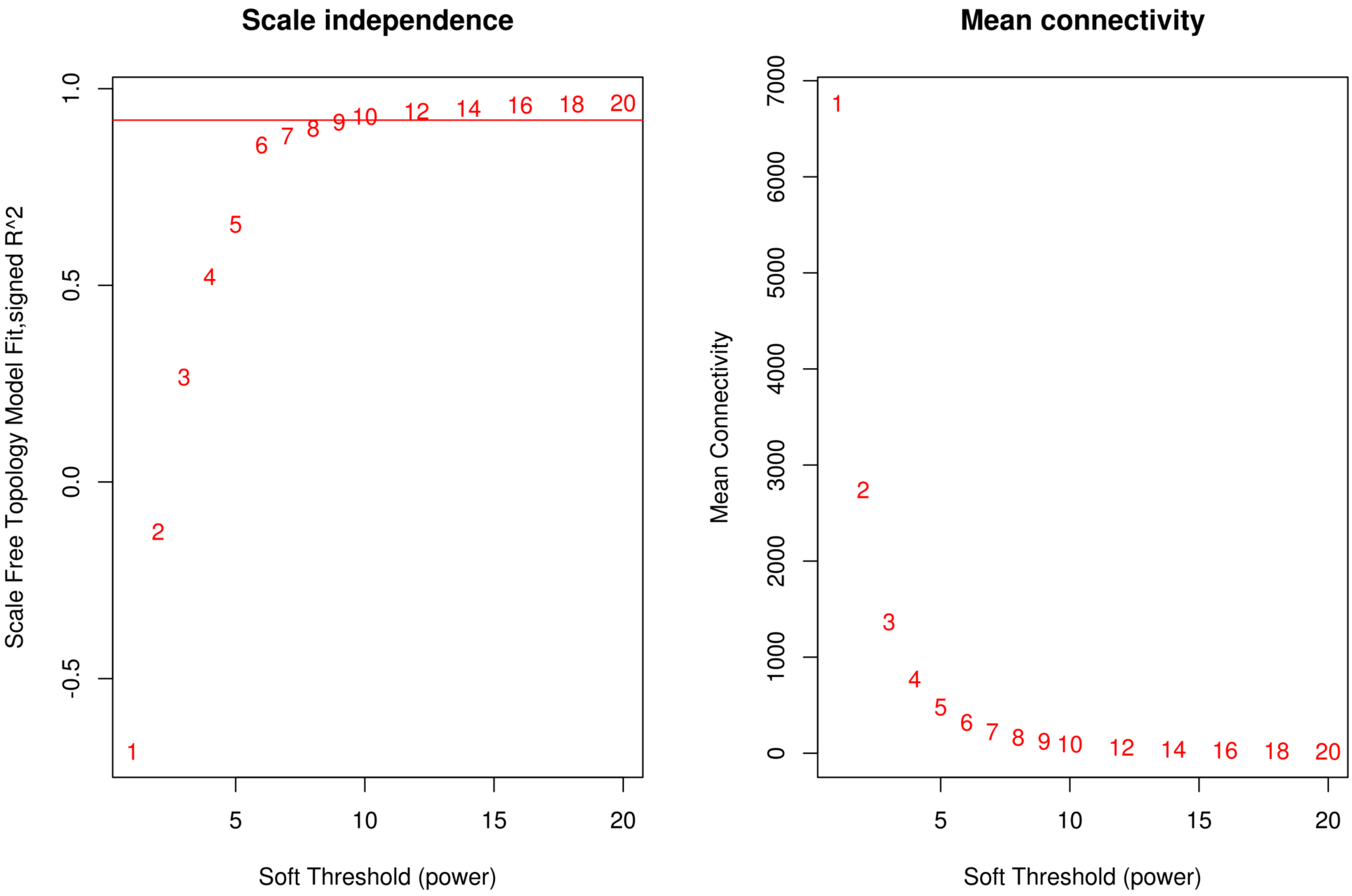


**Supplementary Fig S7:** Evaluation of network topology of Pusa Bold for various soft thresholding powers using pickSoftThreshold function and appropriate power.

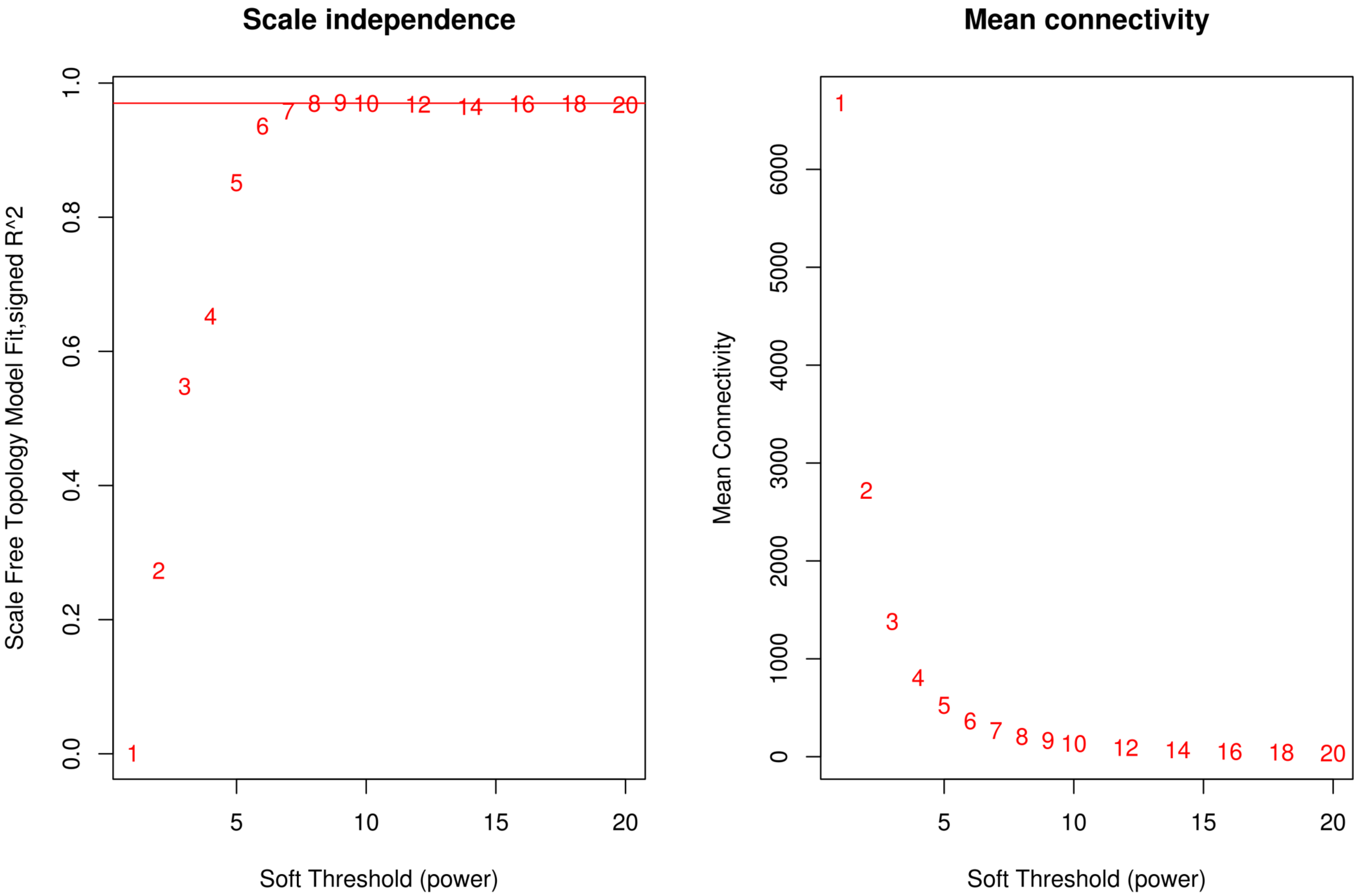


**Supplementary Fig S8:** Evaluation of network topology of Pusa Jaikisan for various soft thresholding powers using pickSoftThreshold function and appropriate power.

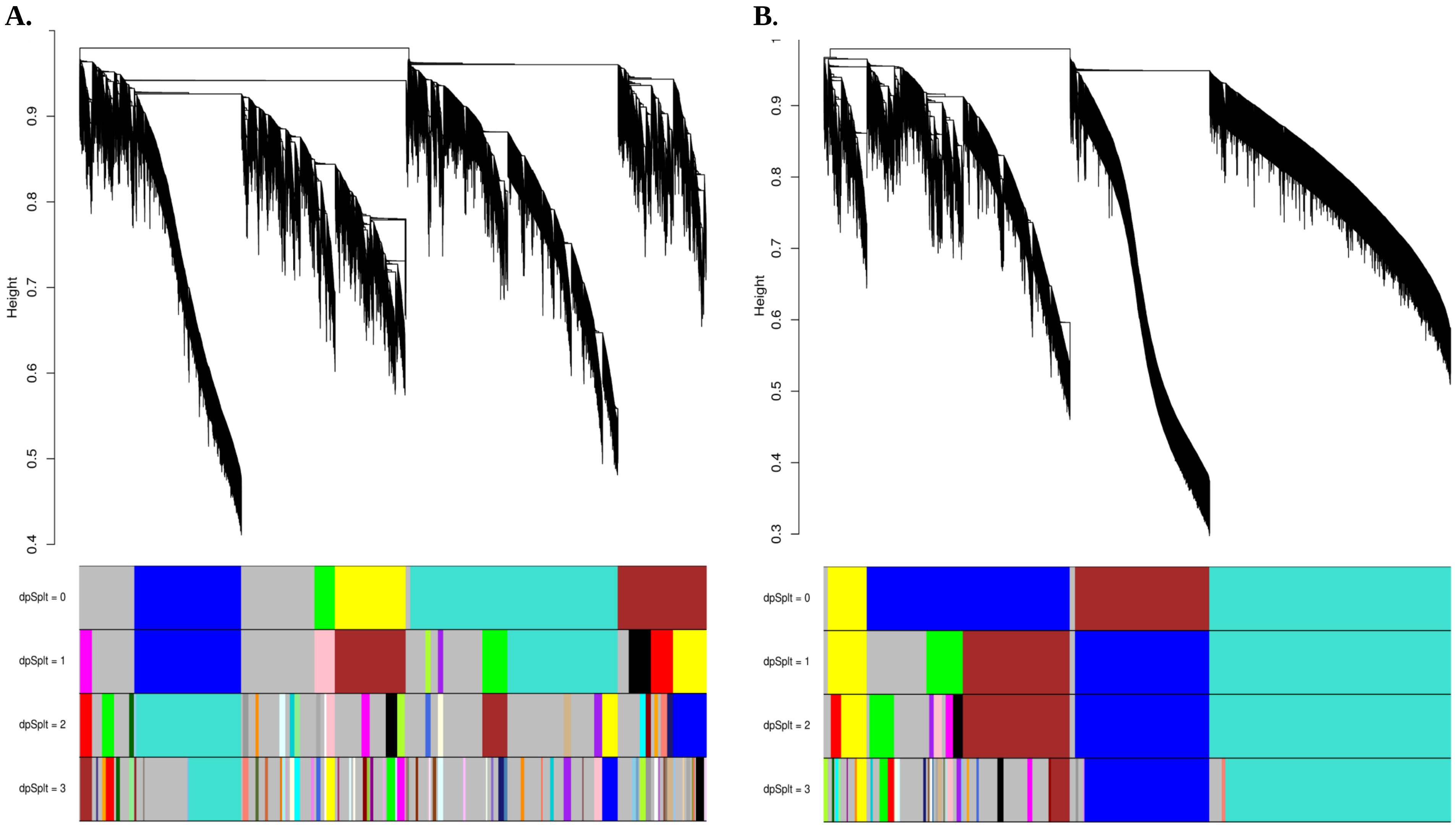


**Supplementary Fig S9:** Hierarchical clustering based on DisTOM: 29 modules in (A) Pusa Bold and 35 modules in (B) Pusa Jaikisan were obtained by deepslit 2 and 3.

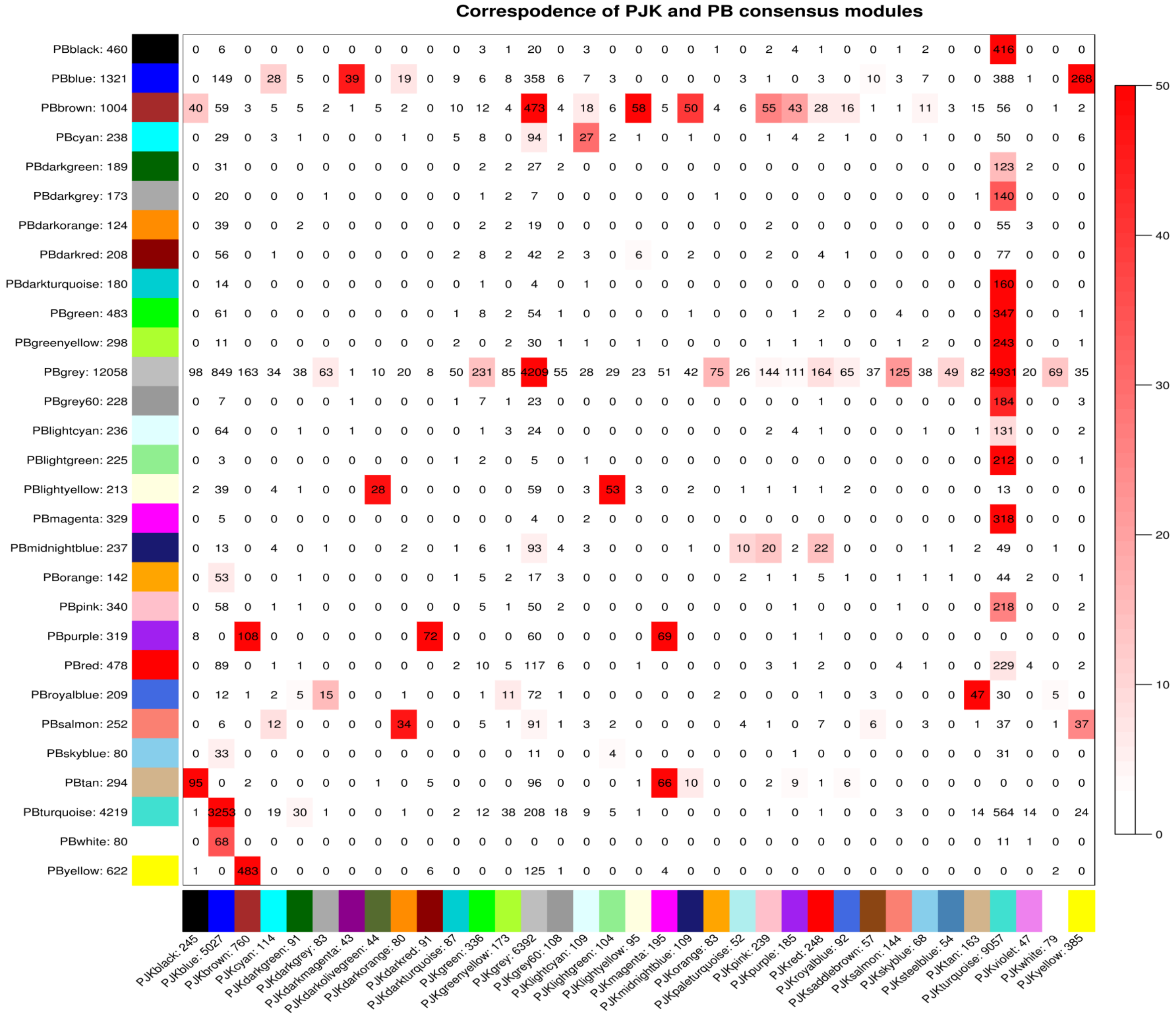


**Supplementary Fig S10: Consensus match of Pusa Bold and Pusa Jaikisan modules:** Modules identifier in the Pusa Bold were compared with most similar module in the Pusa Jaikisan based on significant overlap genes using overlap Table function of WGCNA

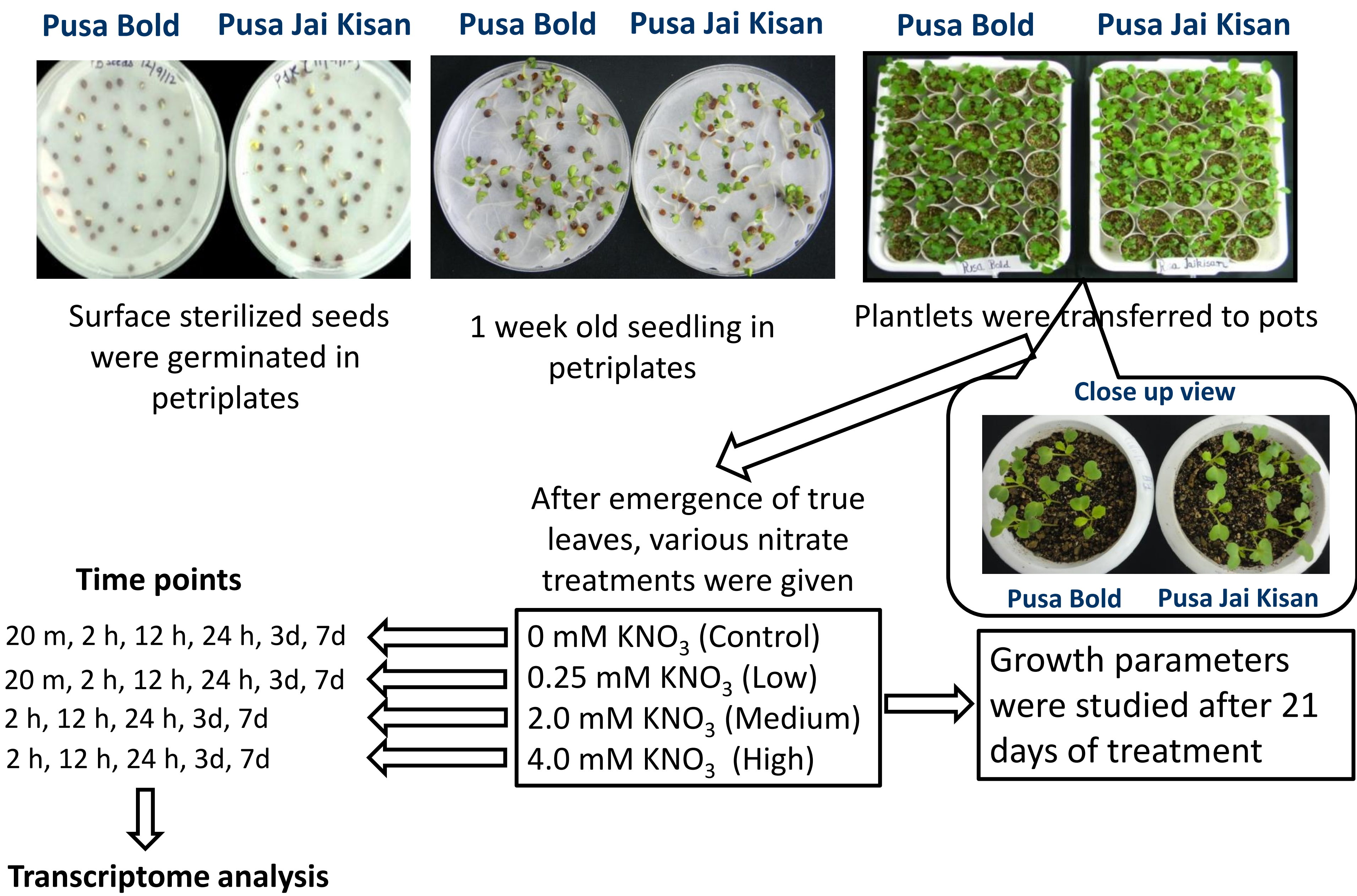


**Supplementary Fig S11:** Experimental design to study growth parameters and transcriptome analysis of Pusa Bold and Pusa Jaikisan in response to various nitrate treatments.

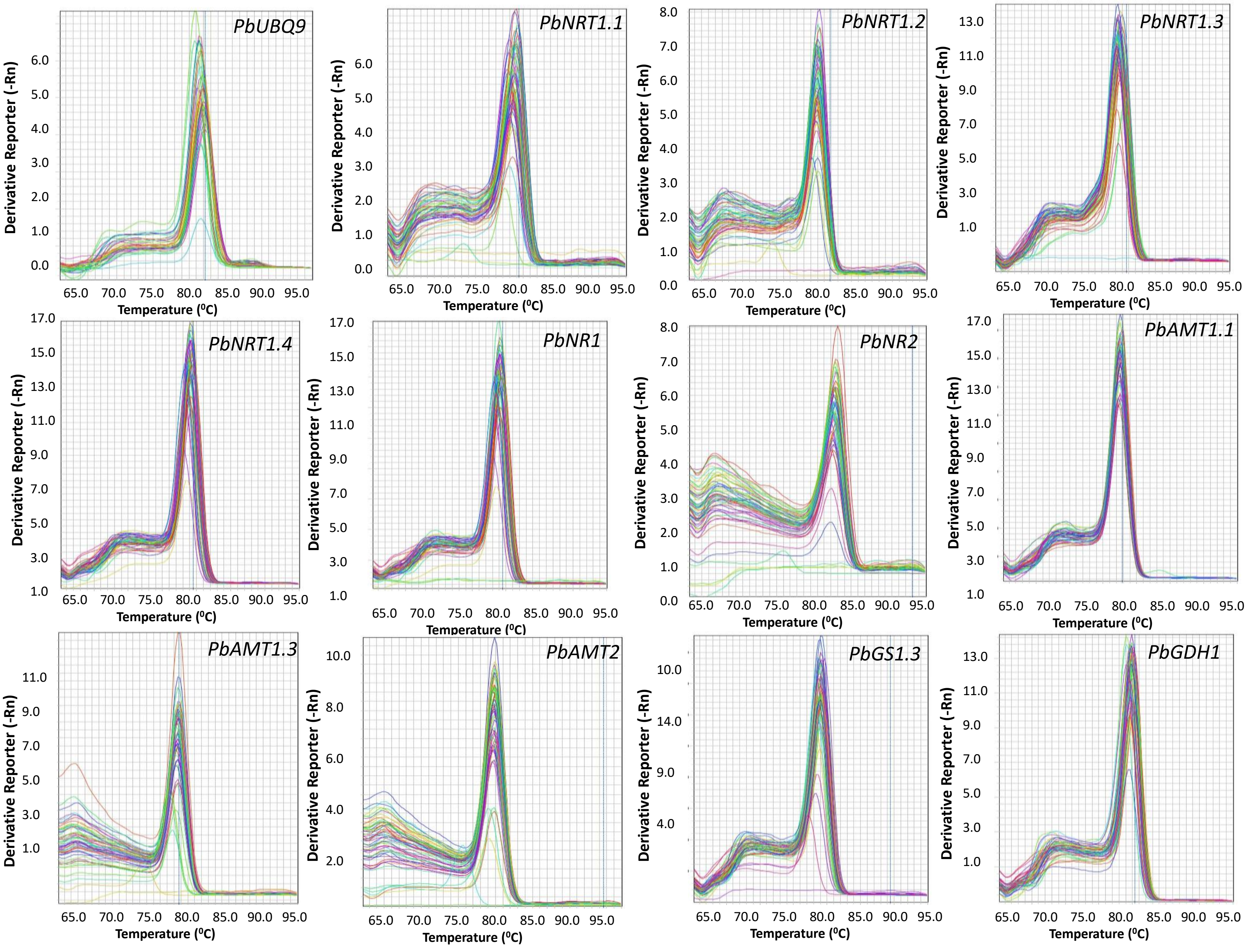


*Continued*

**Supplementary Fig S12:** Melt curves results of all the genes used for qRT-PCR analysis.

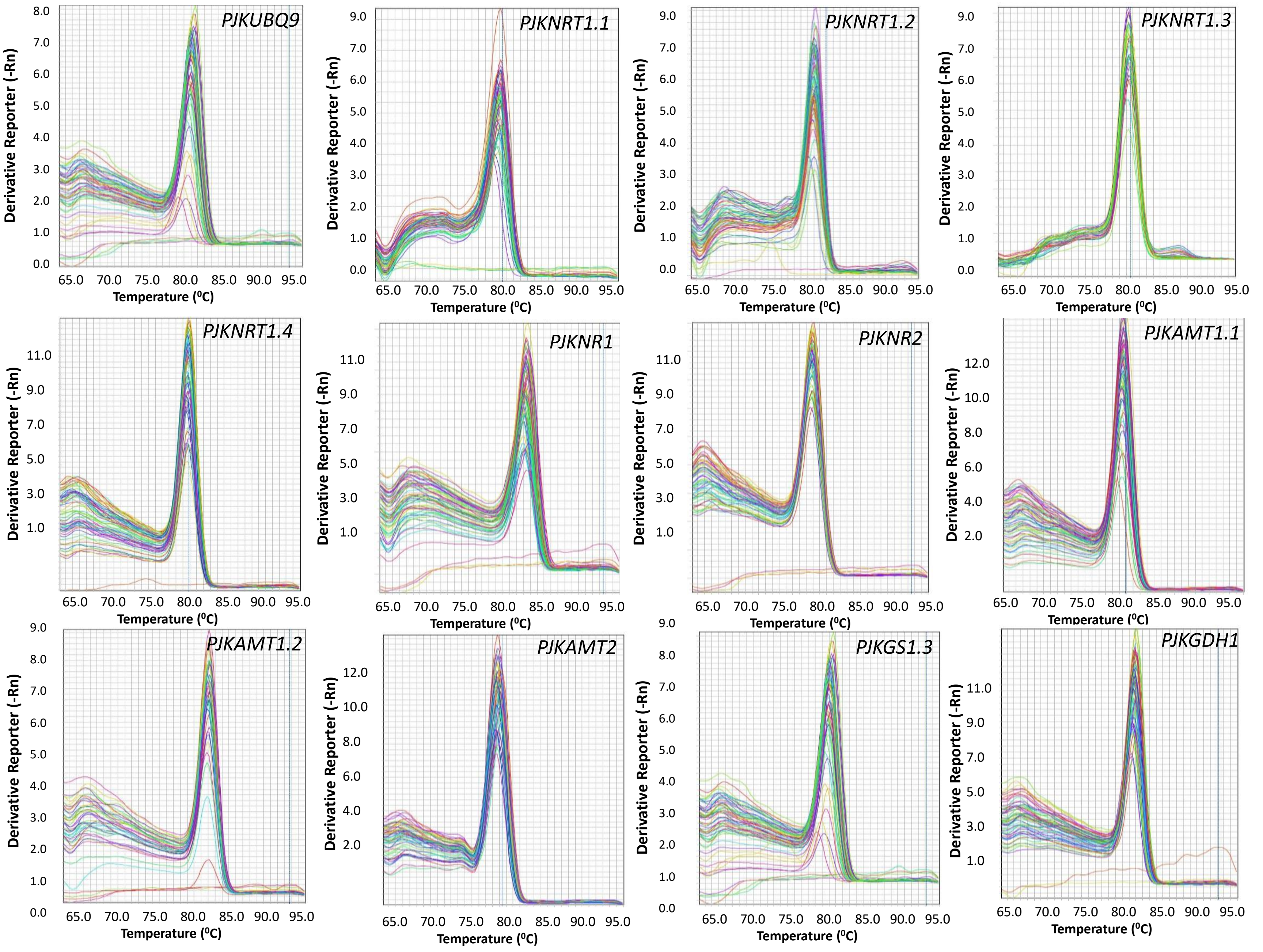


**Supplementary Fig S12:** Melt curves results of all the genes used for qRT-PCR analysis.
